# The SEB-1 Transcription Factor Binds to the STRE Motif in *Neurospora crassa* and Regulates a Variety of Cellular Processes Including the Stress Response and Reserve Carbohydrate Metabolism

**DOI:** 10.1534/g3.116.028506

**Published:** 2016-03-16

**Authors:** Fernanda Zanolli Freitas, Stela Virgilio, Fernanda Barbosa Cupertino, David John Kowbel, Mariana Fioramonte, Fabio Cesar Gozzo, N. Louise Glass, Maria Célia Bertolini

**Affiliations:** * Departamento de Bioquímica e Tecnologia Química, Instituto de Química, Universidade Estadual Paulista (UNESP), 14800-060, Araraquara, São Paulo, Brazil; † Department of Plant and Microbial Biology, University of California, Berkeley, California, 94720-3102; ‡ Departamento de Química Orgânica, Instituto de Química, Universidade Estadual de Campinas (UNICAMP), 13803-862, Campinas, São Paulo, Brazil

**Keywords:** SEB-1, stress response, ChIP-qPCR, RNA-seq, *Neurospora crassa*

## Abstract

When exposed to stress conditions, all cells induce mechanisms resulting in an attempt to adapt to stress that involve proteins which, once activated, trigger cell responses by modulating specific signaling pathways. In this work, using a combination of pulldown assays and mass spectrometry analyses, we identified the *Neurospora crassa* SEB-1 transcription factor that binds to the Stress Response Element (STRE) under heat stress. Orthologs of SEB-1 have been functionally characterized in a few filamentous fungi as being involved in stress responses; however, the molecular mechanisms mediated by this transcription factor may not be conserved. Here, we provide evidences for the involvement of *N. crassa* SEB-1 in multiple cellular processes, including response to heat, as well as osmotic and oxidative stress. The Δ*seb-1* strain displayed reduced growth under these conditions, and genes encoding stress-responsive proteins were differentially regulated in the Δ*seb-1* strain grown under the same conditions. In addition, the SEB-1-GFP protein translocated from the cytosol to the nucleus under heat, osmotic, and oxidative stress conditions. SEB-1 also regulates the metabolism of the reserve carbohydrates glycogen and trehalose under heat stress, suggesting an interconnection between metabolism control and this environmental condition. We demonstrated that SEB-1 binds *in vivo* to the promoters of genes encoding glycogen metabolism enzymes and regulates their expression. A genome-wide transcriptional profile of the Δ*seb-1* strain under heat stress was determined by RNA-seq, and a broad range of cellular processes was identified that suggests a role for SEB-1 as a protein interconnecting these mechanisms.

All living cells are exposed to a range of environmental conditions, which impact their patterns of gene expression, allowing them to adapt to stress conditions and survive. Heat stress results in the activation of specific proteins, known as heat shock transcription factors (HSFs), which bind to DNA regulatory sequences referred to as Heat Shock Elements (HSE) and activate genes that encode heat shock proteins (HSPs). In this process, gene expression is efficiently either up- or down-regulated, resulting in a global cellular adaptation (Sorger and Pelham 1988; Vihervaara *et al.* 2013). In addition to HSEs, the general stress response also includes a different DNA sequence, known as the Stress Response Element or STRE (CCCCT), which was first reported in *Saccharomyces cerevisiae* (Kobayashi and McEntee 1990, 1993). STRE motifs are present in the promoters of genes that are regulated during stress. A search for proteins able to bind to the STRE sequence showed that the zinc finger transcription factor Msn2p, together with its counterpart Msn4p, are the primary STRE-binding proteins in yeast, acting as inducers of the transcription of stress-induced genes (Schmitt and McEntee 1996; Martínez-Pastor *et al.* 1996). About half of the genes induced by stress belong to the Msn2/4p regulon and include oxidative stress, metabolic, and other cytoprotective genes (Gasch 2007; Causton *et al.* 2001; Morano *et al.* 2012). Under normal growth conditions, these transcription factors are cytoplasmic, but translocate to the nucleus when cells are exposed to stress (Görner *et al.* 1998; Jacquet *et al.* 2003).

In *S. cerevisiae*, a large number of stress-responsive genes have STRE motifs in their promoter regions. Although the presence of these motifs frequently correlates with transcriptional activation under stress, the participation of the STRE motif in transcriptional repression has also been described, which is mediated by the action of different transcriptional repressors (de Groot *et al.* 2000; Vyas *et al.* 2005). In *Neurospora crassa*, the *gsn* gene, which encodes glycogen synthase, the regulatory enzyme in glycogen synthesis, is repressed under heat shock (de Paula *et al.* 2002). This regulation may involve the STRE motif located in its promoter region, since DNA-protein complexes have been observed *in vitro* using the *gsn* STREs and proteins from nuclear extracts after exposure of cells to heat stress (Freitas and Bertolini 2004). However, these elements were unable to induce transcription of a reporter gene in *S. cerevisiae* under heat stress, suggesting a repressor role for this *cis* regulatory element in *N. crassa* (Freitas *et al.* 2010).

Filamentous fungi apparently lack Msn2/4p orthologous proteins, which suggests that the regulation of stress responses may have evolved differently among micro-organisms. However, proteins binding to STRE-like sequences have been functionally characterized in a few filamentous fungi and they show similarity and divergence to the well-characterized Msn2/4 yeast proteins, suggesting the existence of distinct molecular mechanisms regulating stress responses in different fungi. In general, these proteins show very little amino acid sequence conservation to the Msn2/4p, only displaying identity in the zinc finger region. One such protein is Seb1 from *Trichoderma atroviride*, which was reported to be involved in, but not essential for, the osmotic stress response (Peterbauer *et al.* 2002; Han and Prade 2002; Seidl *et al.* 2004). This transcription factor failed to complement the Δ*msn2/4* mutant of *S. cerevisiae*, suggesting that it may not be a direct Msn2/4 ortholog (Peterbauer *et al.* 2002). In *Candida albicans*, a Msn2/4p-like transcription factor was initially reported as not being involved in environmental stress responses (Nicholls *et al.* 2004), whereas the *C. glabrata* CgMsn2 protein complemented the *S. cerevisiae msn2* mutant and was required for full resistance against osmotic stress and the induction of genes involved in trehalose synthesis (Roetzer *et al.* 2008).

In addition to their role in stress responses, some putative Msn2/4 homologs have been described as playing a role in virulence and pathogenicity. The *C. glabrata* CgMsn2 protein was required for virulence in a *Drosophila melanogaster* infection model and, more recently, deletion of *sebA*, an ortholog of *seb1* from *Aspergillus fumigatus*, was demonstrated to have a severe impact on virulence and pathogenicity in a murine model (Dinamarco *et al.* 2012). SebA apparently does not play a role in osmotic stress, but is involved in a broader range of stress responses, including the response to heat shock, oxidative stress, and poor nutrient deprivation adaptation (Dinamarco *et al.* 2012).

In this work, we identified by mass spectrometry a transcription factor that binds a STRE motif present in the promoter region of the *gsn* gene in *Neurospora crassa*. This protein is an ortholog of the *T. atroviride* Seb1 transcription factor and was named here as SEB-1. SEB-1 is involved in environmental stress responses such as exposure to heat shock, acidic, osmotic, and oxidative conditions, as the Δ*seb-1* mutant is more sensitive to these stressors than the wild-type strain. The response to stress was correlated with the localization of SEB-1-GFP in the nucleus. In addition, we showed a new role for SEB-1 in the regulation of the metabolism of the reserve carbohydrates glycogen and trehalose. Finally, we used RNA-seq to determine the *seb-1* regulon under heat stress and showed that, in *N. crassa*, the acquisition of heat tolerance is broadly connected with other stress signaling pathways.

## Materials and Methods

### Fungal strains and growth conditions

The *N. crassa* strains FGSC 2489 (A), FGSC 11345 (Δ*seb-1* a), and FGSC 6103 (*his-3* A) were provided by the Fungal Genetics Stock Center (FGSC) (McCluskey *et al.* 2010). A Δ*seb-1* complemented strain (Δ*seb-1 his-3*::*Pccg-1-seb-1-sfgfp*) was constructed in this work. The strain FGSC 6103 was used as female in crosses with the Δ*seb-1* strain to generate a double-mutant strain (Δ*seb-1 his-3*) to be used in complementation experiments. Media and procedures for growth, maintenance, and crosses were as described by Davis and de Serres (1970). The strains were cultivated either on solid or in liquid Vogel’s minimal (VM) medium (Vogel 1956) supplemented, as required, with hygromycin (200 μg/ml) and/or L-histidine chloride (500 μg/ml). Crosses were done by inoculating the opposite mating type strain onto Westergaard’s medium (Westergaard and Mitchell 1947). Transformation and other *N. crassa* molecular techniques were performed as previously described (Colot *et al.* 2006) or using the protocols available at the *Neurospora* homepage (http://www.fgsc.net/Neurospora/NeurosporaProtocolGuide.htm).

For heat shock experiments using liquid cultures, conidia (2 × 10^7^/ml) from the wild-type, Δ*seb-1*, and Δ*seb-1* complemented strains were first germinated in 1 L of VM medium containing 2% sucrose at 30° for 24 hr and 250 rpm. After this, the mycelia were harvested by filtration and a sample was removed, frozen in liquid nitrogen, and stored at –80° until use (control sample). The remaining mycelial pads were transferred to 1 L of fresh VM liquid medium containing 0.5% sucrose preheated at 45° and incubated at 250 rpm for 30 min (heat-shocked samples). The heat-shocked mycelia were harvested by filtration, frozen in liquid nitrogen, and stored at –80° to be further used to determine glycogen and trehalose contents and to prepare total RNA for the RT-qPCR experiments.

### Phenotypic analyses and stress sensitivity

Macroscopic analysis of the wild-type, Δ*seb-1*, and Δ*seb-1* complemented strains was performed on solid VM medium. A volume of 100 μl of a 2 × 10^7^ conidia/ml cell suspension was inoculated in the center of flasks containing solid VM medium and the strains were incubated at 30° for 3 d. After this, they were maintained in daylight at room temperature for 7 d. The 10-day-old cultures were analyzed macroscopically. The colony morphology and hyphal edges were assessed by inoculating 10 μl of a 2 × 10^7^ conidia/ml cell suspension in the center of VM plates followed by incubation at 30° for 24 hr. The images were captured after 24 hr using an AxioCam ICc3 camera coupled to the Zeiss Discovery V8 stereoscope trinocular at 80× magnification. To evaluate the effect of heat stress, the strains were cultivated on solid VM medium at 45° for 48 hr, followed by 24 hr at 30° to check the conidia viability (heat stress recovery).

### Pulldown assay

The pulldown assay was performed to isolate *N. crassa* proteins capable of binding to the STRE motif. The assays used biotinylated oligonucleotides containing the STRE core sequence and NeutrAvidin Plus UltraLink Agarose Resin (Thermo Scientific Pierce Protein Research Products). For this, a nuclear extract was prepared from heat-shocked mycelia (transferred from 30° to 45°) followed by fractionation by affinity chromatography on a Heparin-Sepharose column (GE HealthCare), according to Freitas *et al.* (2008). Chromatographic fractions were individually analyzed by EMSA for their ability to bind to a DNA fragment from the *gsn* promoter containing the STRE motif (STRE1 probe) (Freitas and Bertolini 2004). The fractions showing DNA-binding activity were pooled and used as the protein source for the pulldown assay. The pulldown assay was done as follows: 200 μl of a 50% slurry NeutrAvidin resin was blocked with 500 μg of BSA and 100 μg of denatured salmon sperm DNA in EMSA binding buffer (25 mM HEPES-KOH, pH 7.9; 20 mM KCl, 10% v/v glycerol; 1 mM DTT; 0.2 mM EDTA; 0.5 mM PMSF; 12.5 mM benzamidine; and 5 µg/ml each of antipain and pepstatin A) under agitation for 2 hr at 4°. After blocking, the resin was washed twice with cold EMSA binding buffer and 450 μg of the dsDNA oligo solution (see below) in EMSA binding buffer was added. The mixture was rocked for 2 hr at 4° to couple the oligonucleotides to the avidin-agarose resin. After coupling, 300 µg (total protein) of the active chromatographic fraction was added and the mixture was incubated under mild agitation for 30 min at room temperature. After incubation, the reactions were rapidly washed in cold EMSA binding buffer followed by the addition of 1× Laemmli buffer (Laemmli 1970), boiled, and fractionated on a 12% SDS-PAGE gel. A mock reaction containing the oligonucleotide-free resin was prepared and used as a control. After electrophoresis, the protein bands present in the reaction, but not present in the mock reaction, were excised from the gel, digested with trypsin, and analyzed by mass spectrometry for protein identification.

### Biotinylated DNA oligonucleotides for pulldown assays

Biotinylated double-strand DNA oligonucleotides were prepared as follows and the sequences are shown in Supplemental Material, Table S1. Initially, a pair of complementary oligonucleotides (bioSTRE1-F and STRE1-2R) was designed. The oligonucleotide bioSTRE1-1F was biotinylated and contained two STRE1 core sequences (CCCCT) in tandem with the 5′- and 3′-STRE boundaries, three nucleotides each, acting as a spacer sequence between the two STRE1 motifs. The nonbiotinylated oligonucleotide STRE1-2R was complementary to the bioSTRE1-1F oligonucleotide. A second pair of complementary oligonucleotides (bioSTRE1-1R and STRE1-F) was also designed. The oligonucleotide bioSTRE1-1R was biotinylated and contained one STRE1 core sequence and its 5′- and 3′-boundaries, and was complementary to the oligonucleotide STRE1-F. The two pairs of oligonucleotides were prepared as individual solutions by heating and cooling down slowly to form the dsDNA oligonucleotides bioSTRE1-1F/STRE1-2R and bioSTRE1-1R/STRE1-F. The dsDNA oligonucleotides were quantified and their quality was checked on a 15% nondenaturing PAGE (30:2) in TBE buffer. The individual biotinylated dsDNA oligo solutions were mixed and used as bait in pulldown assays.

### Mass spectrometry analysis

The protein bands excised from the pulldown gels were subjected to ESI-MS/MS analysis to identify the proteins showing STRE-binding activity. All samples were digested with trypsin (Trypsin Gold, Promega) for 15 hr at 37°. Peptides were extracted from the gel with a 50% acetonitrile (ACN)/5% trifluoroacetic acid (TFA) (v/v) solution. The peptide mixture was separated using a BEH C18 column (100 mm × 100 mm - Acquity Waters) with a gradient of 3–70% acetonitrile/water in 0.1% formic acid, using a nanoAcquity UPLC (Waters Co., Manchester, UK) coupled to a Waters Synapt HDMS mass spectrometer. The instrument was operated using the Data Dependent Analysis (DDA) mode, in which the equipment acquires one spectrum per second. When multi-charged species were detected, the three most intense species were fragmented using CID (collision energy defined by the *m/z* ratio and precursor charge). All mass spectra files were converted to a peak list format using Mascot Distiller (Matrix Science) and searched against the *N. crassa* database (http://www.broad.mit.edu/annotation/genome/neurospora/Home.html) using the MASCOT MS/MS ion search tool (http://www.matrixscience.com). The search parameters were as follows: no restrictions on protein molecular weight, one tryptic missed cleavage allowed, nonfixed modifications of methionine (oxidation), fixed modifications of cysteine (carbamidomethylation), with no other posttranslational modifications taken into account. Peptide mass tolerance in searches was 0.1 Da for MS spectra and 0.1 Da for MS/MS spectra. Peptides were considered as identified when their scoring value exceeded the identity or extensive homology threshold value calculated by MASCOT. The sequences of the proteins identified were examined for the presence of domains at the PFAM (http://pfam.janelia.org/) database.

### Cloning and expression of recombinant SEB-1 protein

To clone and express the *seb-1* gene (ORF NCU02671 ), the 1728 bp full-length *seb-1* cDNA sequence was amplified from the *N. crassa* pYADE5 cDNA plasmid library (Brunelli and Pall 1993) using the primers SEB1-F and SEB1-R (Table S1). The entire ORF was inserted into the pGEX-4T1 expression vector (GE HealthCare) leading to the pGEX-SEB1 construction, which was used to transform *Escherichia coli* Rosetta (DE3) pLysS competent cells (Novagen). Cells expressing the GST-SEB-1 protein were cultured in 1 L of LB medium to an OD_600nm_ = 0.8 and protein expression was induced at 12°, 180 rpm for 16 hr using 0.4 mM IPTG (final concentration). Induced cells were harvested and subjected to 10 sonication pulses of 30 sec ON (50% amplitude, ice bath) and 60 sec OFF in phosphate-buffered saline (PBS), pH 7.4 (100 mM Na_2_HPO_4_, 2 mM KH_2_PO_4_, 500 mM NaCl, 2.7 mM KCl, 5% v/v glycerol, 0.5% NP-40 containing 10 mM benzamidine, 0.5 mM EDTA, and 2 mM each of DTT and PMSF) using a Vibra-Cell VCX 750 W cell disrupter (Sonics).

The soluble GST-SEB-1 fusion protein was purified from the crude cellular extract by affinity chromatography (GSTrap HP column, GE HealthCare) on an ÄKTA Purifier purification system, and eluted in 50 mM Tris-HCl (pH 8.0), 20 mM glutathione, 500 mM NaCl, 5% v/v glycerol, and 2 mM DTT. Chromatographic fractions were analyzed by SDS-PAGE on 10% polyacrylamide gels followed by Coomassie Brilliant Blue staining (Laemmli 1970), and the fractions containing the most purified recombinant protein were combined, dialyzed against 10 mM Tris-HCl (pH 7.9), 100 mM KCl, 10% v/v glycerol, 1 mM EDTA, and 0.5 mM DTT, and concentrated. Total protein was determined by the Hartree method (Hartree 1972) using BSA as standard and assayed for DNA-binding activity by EMSA.

### Electrophoretic mobility shift assay (EMSA), DNA probes, and competitors

DNA gel shift experiments were performed according to Freitas and Bertolini (2004). The purified GST-SEB-1 recombinant protein was assayed for DNA-binding activity using, as probes, two 150 bp DNA fragments (STRE1 and STRE2) from the *gsn* gene promoter, both of them containing the STRE motif. The purified recombinant protein GST was used as a negative control in the binding reactions. Binding reactions were carried out in 50 μl of EMSA binding buffer containing 2 µg of the nonspecific competitor poly(dI-dC).(dI-dC) (GE HealthCare) and either 2 or 5 µg of either GST-SEB-1 or GST recombinant proteins. The reaction mixtures were incubated with the radiolabeled probes (∼10^4^ cpm) STRE1 or STRE2, and free probe was separated from DNA-protein complexes on a native 5% polyacrylamide gel. The DNA-protein complexes were detected by autoradiography after exposing the dried gels to X-ray films. For competition assays, an excess of the specific DNA competitors, STRE1 oligonucleotide and either STRE1 or STRE2 cold probe, was added to the binding reactions 10 min prior to incubation with the respective radiolabeled probes.

To prepare the probes and competitors for EMSA, DNA fragments containing the DNA regulatory motifs STRE1 (158 bp) and STRE2 (146 bp) were prepared by PCR according to Freitas and Bertolini (2004) in the absence and presence of [α-^32^P]-dATP (3000 Ci/mmol). The unlabeled STRE1 and STRE2 probes were used as specific competitors for their respective labeled probes. An 18 bp DNA oligonucleotide bearing the STRE1 motif and its boundaries was also used as a specific competitor for both the STRE1 and STRE2 probes after annealing the complementary oligonucleotides STRE1-F and STRE1-R (Table S1). A mutated STRE1 probe (mSTRE1), which changes the STRE1 core sequence CCCCT to AAAAG, was used (Freitas and Bertolini 2004). Probes, specific competitors, and the dsDNA oligonucleotide STRE1 were quantified by measuring the absorbance at 260 nm and added to the reaction in 15-fold excess. The oligonucleotides used for EMSA are listened in Table S1.

### Construction of a Δseb-1 his-3 double mutant and Δseb-1 rescued strains

To complement the Δ*seb-1* mutant, the strain FGSC 11345 (Δ*seb-1*::*hyg* a) was crossed with a *his-3* strain FGSC#6103 (*his-3* A) to generate the double-mutant strain Δ*seb-1 his-3*. A 2023 bp DNA fragment was amplified by PCR with the primer pair sfGFPseb1-F and sfGFPseb1-R (Table S1) using genomic DNA from the wild-type strain as the template. PCR was performed using the Phusion High-Fidelity PCR kit (Finzymes) and DNA fragments were purified with the QIAquick gel extraction kit (Qiagen, CA), according to the manufacturer’s instructions. The purified DNA fragment was cloned into *Xba*I/*Pac*I sites of the plasmid pTSL91-A, resulting in the pTSL91A-*seb-1* construction. The plasmid pTSL91-A allows constitutive expression of *seb-1-sfgfp* from the *ccg-1* promoter. The pTSL91-A-*seb-1* construction was used to transform the Δ*seb-1 his-3* strain, by selection for His+ prototrophy. The transformants (*his-3*::*Pccg-1-seb1-sfgfp*) were selected on VM medium containing hygromicin and/or histidine. The homokaryons were isolated after microconidiation induction (Ebbole and Sachs 1990), followed by filtration using a Millex SV 5 μm (Millipore), and confirmed by PCR using the same primer pair sfGFPseb1-F and sfGFPseb1-R (Table S1).

### Quantification of glycogen and trehalose

Glycogen and trehalose were determined in mycelial pads from the wild-type strain, Δ*seb-1*, and Δ*seb-1* complemented strains submitted or not to heat stress. Briefly, glycogen was precipitated with cold ethanol and digested with α-amylase and amyloglucosidase. Free glucose was quantified with a glucose oxidase kit (Labtest) and the glycogen concentration was normalized to the total protein concentration (Freitas *et al.* 2010). Trehalose content was determined in the same samples according to Neves *et al.* (1991). Briefly, trehalose was digested with a partially purified trehalase from *Humicola grisea* (Zimmermann *et al.* 1990), and the trehalose content was determined by quantifying free glucose using a glucose oxidase kit and normalized to the total protein concentration. Total protein was quantified by the Hartree method (Hartree 1972) using BSA as standard.

### RNA extraction and RT-qPCR analysis

For the RT-qPCR analysis, total RNA from the wild-type strain, Δ*seb-1*, and Δ*seb-1* complemented strains was prepared using mycelial samples subjected or not to heat shock, according to Sokolovsky *et al.* (1990). Expression of the genes *gnn* (glycogenin, NCU06698), *gsn* (glycogen synthase, NCU06687), *gbn* (glycogen branching enzyme, NCU05429), *gpn* (glycogen phosphorylase, NCU07027), *gdn* (glycogen debranching enzyme, NCU00743) related to glycogen metabolism, and the *seb-1* gene was determined by RT-qPCR, using specific oligonucleotides (Table S1). For this, total RNA samples (20 µg) were first treated with RQ1 RNAse-free DNAse (Promega) and subjected to cDNA synthesis using the SuperScript III First Strand Synthesis kit (Invitrogen) and oligo (dT) primer, according to manufacturer’s instructions. The cDNA libraries were subjected to RT-qPCR on a StepOnePlus Real Time PCR system (Applied Biosystems) using the Power SYBR Green PCR Master Mix (Applied Biosystems) and specific primers for each gene amplicon (Table S1). Data analysis was done with the StepOne software (Applied Biosystems) using the comparative CT (ΔΔCT) method (Livak and Schmittgen 2001). Six biological replicates, with three experimental replicates per sample, were performed. The fluorescent dye ROX was used as the passive reference to normalize the SYBR green reporter dye fluorescent signal. The PCR products were subjected to melting curves analysis to verify the presence of single amplicons. All reaction efficiencies varied from 94 to 100%. The actin gene (*act* gene, NCU04173) was used as the reference gene.

### Subcellular localization of SEB-1-sfGFP protein

To determine the subcellular localization of the fluorescent SEB-1-sfGFP protein under heat stress, 200 μl of a conidial suspension (2 × 10^6^ conidia/ml) from the Δ*seb-1* complemented strain were inoculated onto coverslips, covered with VM liquid plus 1% sucrose, and incubated at 30° for 10 hr. After incubation, the coverslips were transferred to fresh VM media containing 0.5% sucrose preheated to 45° and incubated at the same temperature for up to 2 hr. For osmotic stress, the coverslips were shifted to fresh VM media containing 0.5% sucrose, and either 1.5 M sorbitol or 1.5 M NaCl, and incubated at 30° for up to 4 hr. To analyze oxidative stress, the coverslips were shifted to fresh VM liquid media containing 0.5% sucrose and either 500 μM paraquat, 100 μM menadione, or 25 mM hydrogen peroxide, and incubated at 30° for up to 2 hr. For nuclei analysis, mycelia were fixed [3.7% formaldehyde, 50 mM NaH_2_PO_4_ (pH 7.0), 0.2% (v/v) Tween 80], washed twice with PBS and stained with 100 µl DAPI (4’,6-diamidino-2-phenylindole, 0.5 mg/ml) for 5 min. Fluorescence was visualized using a fluorescence microscope with excitation and emission wavelengths of 359 nm and 461 nm, respectively. The images were captured using an AXIO Imager.A2 Zeiss microscope coupled to an AxioCam MRm camera and processed using AxioVision software, version 4.8.2.

### cDNA libraries preparation and RNA-seq

Total RNA was extracted from wild-type and Δ*seb-1* heat-shocked mycelial pads as previously described (Sokolovsky *et al.* 1990), quantified in a NanoDrop ND1000 spectrophotometer (Thermo Scientific), and checked for integrity of rRNA by electrophoresis on a 1.2% formaldehyde agarose gel. The mRNA from biological triplicates was isolated from total RNA samples using Dynabeads Oligo(dT)25 (Invitrogen) and fragmented by the RNA Fragmentation Reagents kit (Ambion). The mRNA fragments were precipitated with 0.1 vol of 3 M sodium acetate and 2 vol of cold 100% ethanol, and washed with 70% ethanol. The precipitate was resuspended in Tris-EDTA buffer. The first cDNA strand was prepared using the SuperScript III First Strand Synthesis kit (Invitrogen) and [d(N_6_)] random primers (Invitrogen), and the second cDNA strand was prepared using the Second Strand Buffer (Invitrogen), according to the manufacturer’s instructions.

For the RNA-seq experiments, the double-stranded cDNAs were end-labeled with different adaptors using the TruSeq DNA LT sample prep kit (Illumina), according to the manufacturer’s instructions. The end-labeled cDNAs of about 200 bp were purified by electrophoresis on a 2.5% low-melting point agarose gel (Qiagen, CA). The gel-purified cDNA libraries were amplified by PCR (TruSeq v2 LT sample prep kit PCR, Illumina), quantified, and checked for quality on an Agilent 2100 Bioanalyzer, and sequenced as single-end 50 bp reads on an Illumina Genome Analyzer GX platform.

### RNA-seq data analysis

The short reads were aligned to the published genome of *N. crassa* (http://www.broadinstitute.org/annotation/genome/neurospora/MultiDownloads.html) using TopHat v2.04 software (Trapnell *et al.* 2009). Cufflinks v2.02 software (Trapnell *et al.* 2010) was used to normalize the reads as fragments per kb or exon model per million mapped fragments (FPKM) for each gene identified. The statistical significance of differences in FPKM among the samples was determined using the CuffDiff component of the Cufflinks package. Only adjusted *P*-values below 0.05 were used to identify significant differences in gene expression before and after heat stress. DEseq v1.14 was utilized for clustering the whole RNA-seq data sets by a Euclidean distance matrix using a variance-stabilizing transformation (Anders and Huber 2010). The retrieved ORFs were functionally annotated and analyzed using the web tool Blast2GO version 2.7.2 (http://www.blast2go.com/start-blast2go) (Conesa *et al.* 2005).

### ChIP-qPCR analysis

Chromatin immunoprecipitation (ChIP) assays were performed on mycelial samples from the Δ*seb-1* complemented strain according to Tamaru *et al.* (2003), with modifications described in Cupertino *et al.* (2015). Briefly, chromatin from mycelia submitted or not to heat shock was fixed with formaldehyde and suspended in ChIP lysis buffer (50 mM HEPES, pH 7.9; 90 mM NaCl; 1 mM EDTA, pH 8.0; 1% Triton X-100; 0.1% sodium deoxycholate; 1 mM PMSF; 0.1 mM TCLK; 1 mM benzamidine; and 1 μg/ml each of pepstatin and antipain). The chromatin was sheared to an average size of 0.3–0.8 kb using Vibra Cell Sonics (10 cycles: 1 min, 40% amplitude, 8.0 sec ON, 9.9 sec OFF). Sonicated chromatin was precleared with Dynabeads Protein A (Novex) preblocked with 0.5% BSA in PBS, and then immunoprecipitated with anti-GFP antibody (Sigma) and Dynabeads Protein A. The DNA concentration was quantified and 25 ng of input DNA (positive control), no Ab, and IPs (immunoprecipitated DNAs from anti-GFP) was analyzed by qPCR on the StepOnePlus Real-Time PCR system (Applied Biosystems) using the Power SYBR Green PCR Master Mix (Applied Biosystems). Oligonucleotides for the *gsn* (emsaSTRE1-F/emsaSTRE1-R and STRE2i-F/STRE2i-R), *gpn* (pGPNNit-F2/pGPNNit-R2), *gnn* (GNNp-F2/GNNp-R3), *gbn* (BRANCH-FP3/BRANCH-RP1), and *gdn* (DEBp-F2/DEBp-R2) promoters are described in Table S1. A ubiquitin gene fragment (NCU05995), which does not have the SEB-1 motif, was amplified with the primers qUbi-F/qUbi-R and used as a negative control of binding. The amplifications were carried out in triplicates in a 96-well plate. All PCR products had melting curves indicating the presence of a single amplicon. For each promoter region analyzed, at least five independent standard curves were run and the mean cycle threshold value at each 10-fold dilution of three independent runs was used for the final standard curve.

### Data availability

Strains and plasmids can be provided upon request. File S1 contains detailed descriptions of all supplemental files.

## Results

### Identification of SEB-1 as a transcription factor that binds to STRE in N. crassa

**Figure 1:**
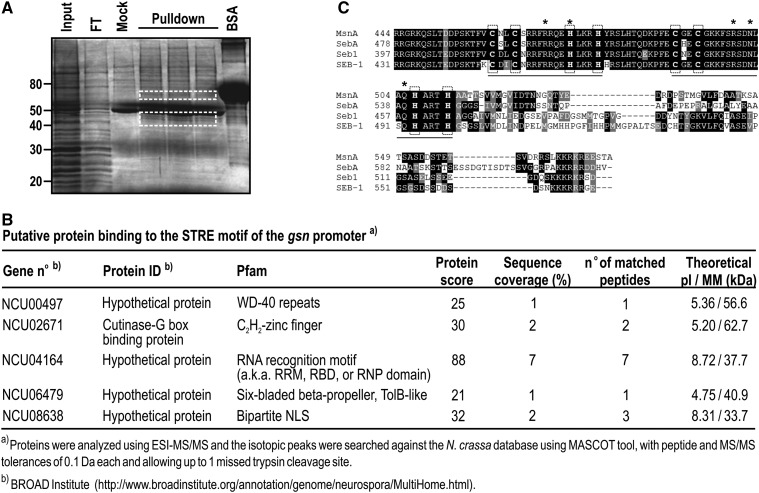
Pulldown assay identified SEB-1 as the transcription factor that binds to the STRE motif in *N. crassa*. (A) Biotin-streptavidin pulldown assay. Biotinylated dsDNA oligonucleotides containing the *N. crassa* STRE consensus sequence in tandem were used as bait in a pulldown reaction with nuclear extract prepared from heat-shocked mycelia as a protein source. Protein bands not present in the mock reaction were removed from the gel, trypsin digested and submitted to mass spectrometry (white rectangles). Input: protein fraction showing DNA-binding activity; Mock: pulldown reaction without dsDNA coupled to the resin; FT: flow-through chromatographic sample. (B) The putative proteins identified by mass spectrometry. Proteins were analyzed by ESI (electrospray ionization)-MS/MS. Monoisotopic peaks were searched against the *N. crassa* database with a maximum of one missed trypsin cleavage and a mass tolerance of 0.1 Da for precursor ions and 0.1 Da for MS/MS spectra. Database was searched by using MASCOT. (C) Sequence alignment of different Msn2p/4p orthologs present in filamentous fungi. Alignment was done using Delta-BLAST (BLASTP 2.2.29+) in a nonredundant database. MsnA: *A. nidulans* ortholog (XP659256.1); SebA: *A. fumigatus* ortholog (XP751917.1); Seb-1: *T. atroviride* ortholog (AAM73769.1); SEB-1: *N. crassa* ortholog (EAA36208.2). The bars indicate the Zinc finger domains, and the cysteine and histidine residues are highlighted in vertical rectangles (dotted lines). The putative amino acid residues interacting with the STRE motif are indicated by asterisks. BSA, bovine serum albumin; STRE, stress response element.

In *S. cerevisiae*, STRE is a motif present in the promoters of stress-regulated genes and is recognized by the transcription factors Msn2p/4p (Martínez-Pastor *et al.* 1996). The *GSY2* gene in *S. cerevisiae*, which encodes an isoform of glycogen synthase, has STRE in its promoter and is activated under heat stress (Ni and LaPorte 1995). However, the gene encoding the same enzyme (*gsn*) in *N. crassa* is down-regulated under heat stress (de Paula *et al.* 2002), although it has STRE motifs in its 5′-flanking region (Freitas and Bertolini 2004), suggesting that the stress response mediated by STRE may differ between yeast and *N. crassa*. A search for Msn2p/4p orthologs in the *N. crassa* genome retrieved no significant results, suggesting that *N. crassa* may lack orthologs of these transcription factors. We used pulldown assays with biotinylated primers and cellular extracts from heat-shocked cells to isolate proteins able to bind STRE, and to identify the proteins by mass spectrometry. Oligonucleotides containing tandem repeats of the STRE core sequence and genomic nucleotide sequences as spacer regions (Table S1) were coupled to streptavidin beads, and a chromatographic fraction showing DNA-binding activity was used as the protein source. The proteins were eluted from the resin and separated on a SDS-PAGE gel, revealing the presence of two regions in the gel containing protein bands that were not present in the mock reaction (Figure 1A, compare the mock lane with pulldown lanes). These regions were excised from a SDS gel, trypsin digested, and submitted to mass spectrometry analysis for protein identification. Of the proteins identified in the pulldown assays (Figure 1B), one protein had a double C_2_H_2_-type zinc finger DNA-binding domain at its C-terminus – the protein encoded by the ORF NCU02671. This ORF is annotated in the *N. crassa* database as encoding a cutinase G-box binding protein, containing 575 amino acid residues with theoretical molecular mass and pI values of 62.7 kDa and 5.2, respectively. The two zinc finger motifs extend from amino acid residues 448–497 (Figure 1C). A Delta-BLAST analysis showed that the protein was a homolog of the Seb1 from *T. atroviride* (40% identity) (Peterbauer *et al.* 2002), MsnA from *A. nidulans* (34% identity) (Han and Prade 2002), and SebA from *A. fumigatus* (31% identity) (Dinamarco *et al.* 2012) proteins. The identities are mainly localized in the C-terminus, where the two zinc finger motifs are located (Figure 1C). According to PSORT II (http://psort.hgc.jp/form2.html), the protein has three monopartite nuclear localization signals at amino acid positions 363 (PSKRARH), 432 (PTNRRGR), and 568 (KKKR). The Seb-1, MnsA, and SebA proteins are transcription factors that are responsive to stress conditions in filamentous fungi. In addition, the *T. atroviride* Seb1 transcription factor binds *in vitro* to the *S. cerevisiae* STRE core sequence 5′-AGGGG-3′ (Peterbauer *et al.* 2002). Therefore, we named NCU02671 as *seb-1* and predicted that SEB-1 protein may be a transcription factor that binds to the STRE motif in *N. crassa*.

### The seb-1 deletion causes morphological defects and sensitivity to stress

**Figure 2:**
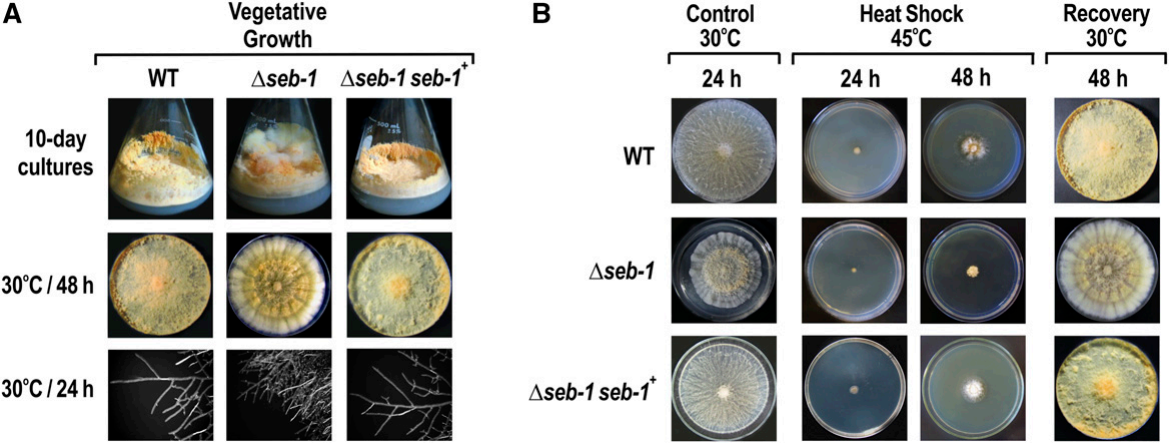
Functional characterization of the mutant strain Δ*seb-1*. (A) 10-day-old conidia of the Δ*seb-1* strain were cultivated in Erlenmeyer flasks and on plates containing solid VM medium. The images of the hyphae tips were captured after 24 hr using an AxioCam ICc3 camera coupled to a trinocular Discovery V8 stereoscope (Zeiss) at 80× magnification. (B) Heat stress assay. The Δ*seb-1* strain was cultivated at 45° for 2 d and then brought back to 30° for 24 hr to evaluate cellular viability. Cultures at 30° for 24 hr are shown as control. The Δ*seb-1 seb-1*^+^ strain is the Δ*seb-1* complemented strain (Δ*seb-1 his-3*::*Pccg-1-seb1-sfgfp*). VM, Vogel’s minimal; WT, wild-type.

To assess the effects of the loss of SEB-1 function, we examined the morphology of the Δ*seb-1* strain during vegetative growth. Flasks of 10-day-old cultures of the Δ*seb-1* strain exhibited a cauliflower-like growth aspect, as compared to the wild-type strain (Figure 2A). The radial growth of the Δ*seb-1* mutant showed the presence of growth bands with different regions of pigmentation (Figure 2A). Analysis of the hyphal edges of the Δ*seb-1* mutant revealed numerous short hyphae, most likely resulting from a hyper-branching phenotype (Figure 2A). To confirm that the morphological changes were due to the *seb-1* knockout, we constructed the Δ*seb-1* complemented strain by inserting the *seb-1* gene from the wild-type strain into the *his-3* locus (Δ*seb-1 his-3*::*seb-1*). When cultured under the same conditions as the mutant and wild-type strains, the rescued strain (Δ*seb-1 his-3*::*seb-1*) restored the wild-type growth phenotype, confirming that the morphological aspects observed in the mutant strain were indeed due to the *seb-1* deletion (Figure 2A).

To investigate the function of the SEB-1 protein in stress responses, we evaluated the sensitivity of the Δ*seb-1* and Δ*seb-1* complemented strains to heat and pH changes and to a variety of chemical stresses, such as exposure to high concentrations of sorbitol, NaCl, paraquat, menadione, and hydrogen peroxide, and compared the results with those of the wild-type strain. We first analyzed the radial growth of the mutant strain after 24 and 48 hr of incubation at 45°. Radial growth at 30° for 24 hr was lower than those observed in the wild-type and complemented strains. Compared to the wild-type strain, Δ*seb-1* showed defective growth after 48 hr of heat stress (Figure 2B). The response to heat shock mediated by SEB-1 was confirmed by analyzing the Δ*seb-1* complemented strain under the same condition; the rescued strain showed similar growth to the wild-type strain (Figure 2B). The viability of the heat-shocked cells was evaluated by shifting the 48 hr cultures back to 30° for 24 hr. All of the strains recovered their normal morphological aspects, confirming that the growth response to high temperature was due to viable cells (Figure 2B).

The pH stress response was analyzed by comparing the growth of the strains under acidic (4.2) and alkaline (7.8) pH with growth under pH 5.8 (normal pH) for 24 and 48 hr. The Δ*seb-1* strain was sensitive to both pH, exhibiting lower radial growth than the wild-type and rescued strains, and high sensitivity at pH 7.8 (Figure S1). The Δ*seb-1* strain exhibited a dense growth halo under pH 4.2 after 48 hr, which consisted of thin, highly vacuolated hyphae, suggesting that this halo corresponded to a zone of cellular death (results not shown).

Oxidative stress in the Δ*seb-1 vs.* wild-type and rescued strains was evaluated by cultivating the strains in liquid and solid VM medium in the presence of increasing concentrations of the ROS-forming agents paraquat, menadione, and hydrogen peroxide. These oxidant agents orchestrate different responses that converge to a same final product, *i.e.*, the generation/accumulation of ROS within the living cells. Paraquat is a viologen herbicide that interferes with the respiratory chain, accepting electrons from a donor such as NADPH and transferring them to molecular oxygen, thereby producing high amounts of the ROS superoxide anion (O_2_^–^). On the other hand, menadione (or vitamin K3) is a synthetic naphthoquinone derivative that is reduced to its hydroquinone form in a NADPH-dependent way. An excess of menadione causes NADPH depletion and glucose-6-phosphate dehydrogenase (G6PD) deficiency. As a consequence of NADPH decay, there is no supply of reduced glutathione, which leads to increased levels of intracellular hydrogen peroxide. The Δ*seb-1* strain showed high sensitivity to all oxidative stress agents, both in liquid and solid cultures (Figure S2, compare A and B), mainly at the lower concentrations used in this assay (see right panel in Figure S2A).

The tolerance to osmotic stress was analyzed by growing the strains in the presence of increased concentrations of sodium chloride and sorbitol in solid and liquid VM medium. The Δ*seb-1* strain was highly sensitive to both high salt and high carbohydrate concentrations (Figure S3, A and B). The sensitivity of the mutant strain was also higher under lower concentrations of sodium chloride and sorbitol (see right panel in Figure S3A). The rescued strain exhibited a phenotype similar to the wild-type strain in all stress conditions analyzed here, confirming that the increased sensitivity to stress was indeed caused by the lack of the SEB-1 transcription factor.

### SEB-1 cellular localization under different environmental conditions

**Figure 3:**
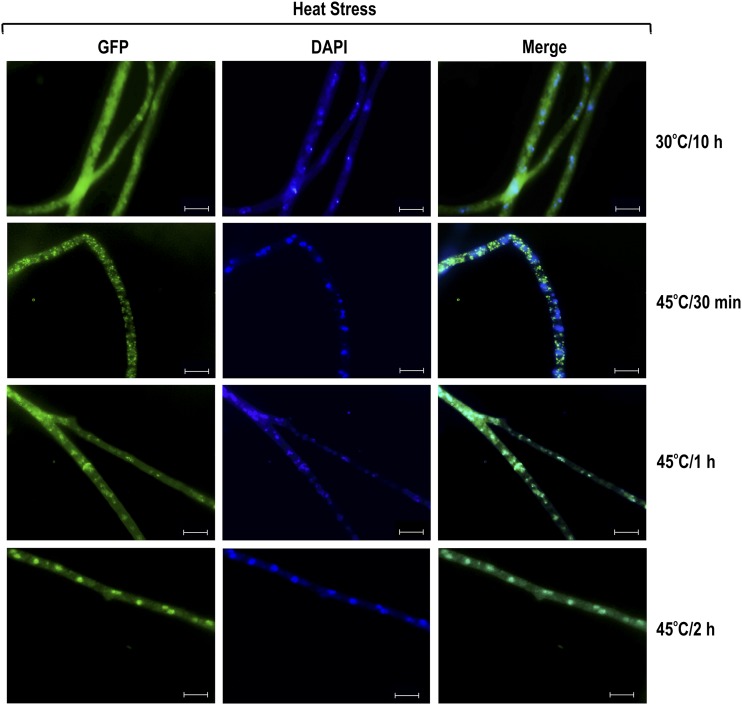
SEB-1 protein translocates to the nucleus during heat stress. Conidia from the Δ*seb-1* complemented strain (Δ*seb-1 his-3*::*Pccg-1-seb1-sfgfp*) were grown on coverslips in liquid VM medium containing 1% sucrose at 30° for 10 hr. After this period, mycelia were transferred to VM medium preheated at 45° (heat stress) for 30 min, 1 hr, and 2 hr. Heat-shocked mycelia were fixed in PBS with formaldehyde, the nuclei were stained with DAPI (0.5 mg/ml), and the fluorescence was examined. Mycelium cultured in liquid VM medium at 30° for 10 hr was used as a control. Images were taken after 30 min, 1 hr and 2 hr after transferring to 45°. Fluorescence was evaluated using the AXIO Imager.A2 microscope (Zeiss) at a magnification of 100×. The images are representative of at least two independent experiments. Scale bar: 10 μm. DAPI, 4’,6-diamino-2-phenylindole; GFP, green fluorescent protein; PBS, phosphate-buffered saline; VM, Vogel’s minimal.

**Figure 4:**
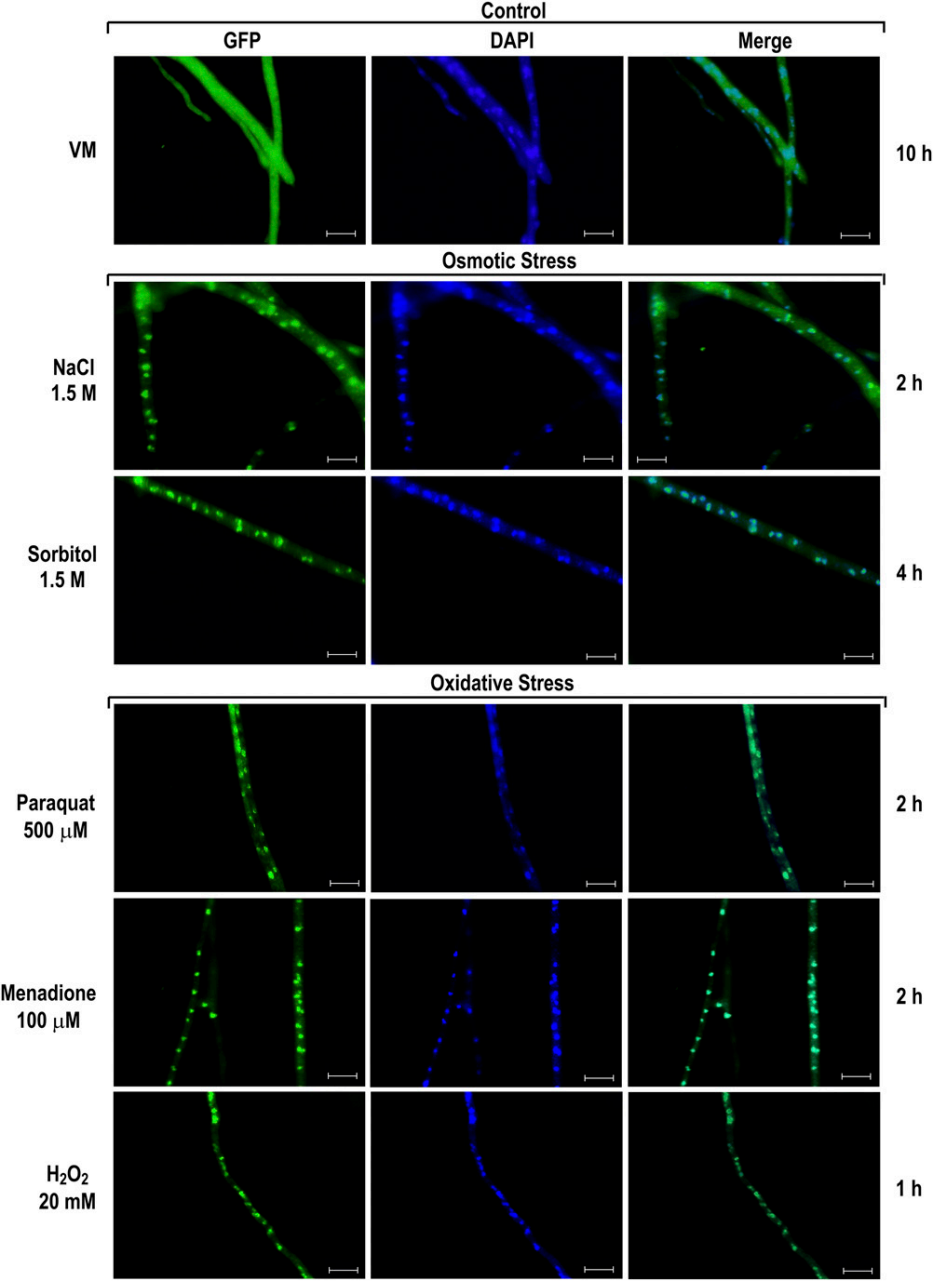
SEB-1 protein translocates to the nucleus under osmotic and oxidative stress conditions. Conidia from the Δ*seb-1* complemented strain (Δ*seb-1 his-3*::*Pccg-1-seb1-sfgfp*) were grown on coverslips in liquid VM medium containing 1% sucrose at 30° for 10 hr. After this period, mycelia were subjected to osmotic and oxidative stresses for 1 hr, 2 hr, or 4 hr. Stressed mycelia were fixed in PBS with formaldehyde, the nuclei were stained with DAPI, and the fluorescence were visualized. Mycelia from liquid VM medium cultured for 10 hr at 30° was used as control. Fluorescence was evaluated using the AXIO Imager.A2 microscope (Zeiss) at a magnification of 100×. The images are representative of at least two independent experiments. Scale bar: 10 μm. DAPI, 4’,6-diamino-2-phenylindole; GFP, green fluorescent protein; PBS, phosphate-buffered saline; VM, Vogel’s minimal.

The functionality of SEB-1 was also evaluated by determining its subcellular localization under different stress conditions. To assess this, we used the Δ*seb-1* complemented strain, in which SEB-1 is produced as a C-terminus GFP-tagged fusion protein. We first evaluated the SEB-1 cellular localization under heat stress by germinating conidia at 30° for 10 hr and then transferring to medium at 45°. The SEB-1-GFP protein was found to be predominantly in the cytoplasm after 30 min of heat stress, but was translocated to the nucleus after 2 hr of exposure to 45° (Figure 3). These data are consistent with its role in regulating the heat stress response in *N*. *crassa*. The cellular localization of SEB-1-GFP was also analyzed under osmotic and oxidative stress. For osmotic stress, *N. crassa* conidia were germinated in glucose VM medium for 10 hr and then transferred to VM medium containing either NaCl or sorbitol. In both conditions, SEB-1-GFP was mainly detected in the nucleus, but some cytoplasmic localization was observed even 4 hr after transfer (Figure 4). The cellular location of SEB-1-GFP was also evaluated under oxidative stress induced by paraquat, menadione, or H_2_O_2_. All chemicals caused translocation of SEB-1-GFP to the nucleus, although the protein was also observed in the cytoplasm (Figure 4). Therefore, the *N. crassa* SEB-1 transcription factor cycles between the cytoplasm and the nucleus under environmental situations that promote stress.

### The SEB-1 transcription factor binds to STRE in vitro

**Figure 5:**
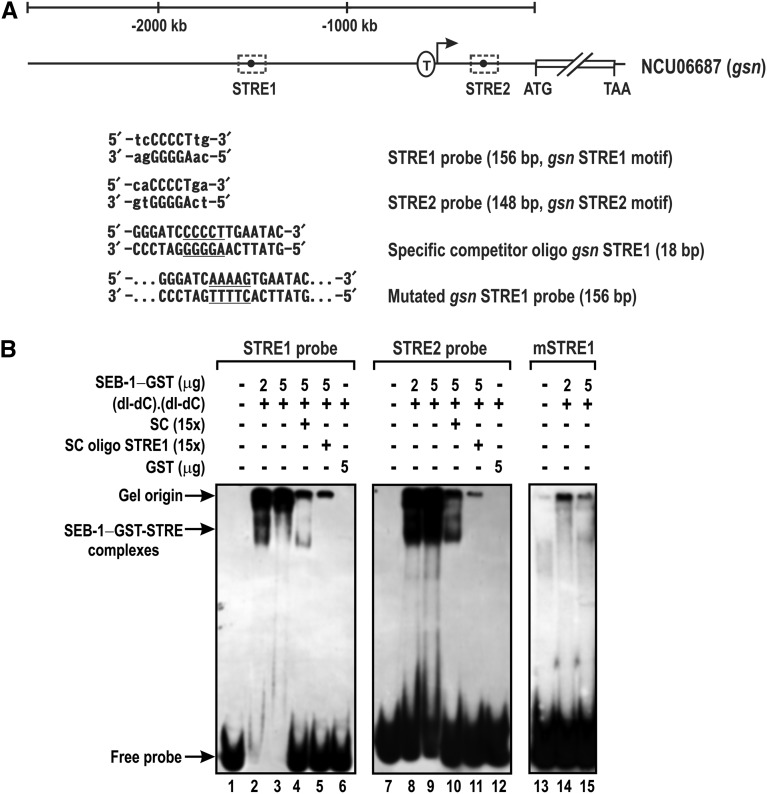
Recombinant SEB-1 binds specifically to the *N. crassa* STRE motif *in vitro*. (A) Schematic representation of the *gsn* gene and the STRE positions in the promoter region. The sequences of the STRE1 and STRE2 DNA elements are represented. The STRE1 DNA oligonucleotide containing the motif sequence (underlined) and the STRE1 probe with mutations in the STRE core sequence (underlined) are also represented. T: transcription start site (Freitas and Bertolini 2004). (B) DNA probes containing both STRE motifs present at the 5′-flanking region of the gene *gsn* (STRE1 and STRE2) were assayed for DNA-binding activity using the GST-tagged recombinant SEB-1 as a protein source. The specificity of the DNA shifts was demonstrated by the prior addition of specific competitors: the cold probes STRE1 and STRE2, the dsDNA oligonucleotide STRE1, and the dsDNA fragment containing the mutated STRE1 motif. SC: specific competitor (unlabeled probes STRE1 and STRE2); SC oligo STRE1: specific competitor corresponding to a short dsDNA oligonucleotide (18 bp) containing the STRE1 motif; mSRE1: probe STRE1 containing the mutated STRE1 motif. dsDNA, double-stranded; GFP, green fluorescent protein; STRE, stress response element.

As previously described, SEB-1 was identified using an approach to isolate a transcription factor capable of binding to the STRE motif present in the promoter of the gene encoding glycogen synthase (*gsn*), the rate-limiting enzyme in glycogen synthesis. This gene has two STRE motifs in its 5′-flanking region (Figure 5A), the first one located within the promoter region upstream of the putative TATA-box (STRE1), and the other one within the intron located in the 5′-UTR (STRE2) (Freitas and Bertolini 2004). To confirm that SEB-1 binds to STRE in *N. crassa*, the GST-tagged recombinant protein was produced in *E. coli* and used as a protein source for EMSA using two DNA fragments of the 5′-flanking region, one bearing STRE1 (probe STRE1) and the other one bearing STRE2 (probe STRE2). The gel shift assay showed that the GST-SEB-1 fusion protein bound to both motifs *in vitro* (Figure 5B, lanes 2, 3, 8, and 9). The binding specificity was confirmed for both DNA probes since the specific competitors (the cold probes STRE1 and STRE2), strongly decreased the shifted bands when 5 μg of the recombinant protein was used (Figure 5B, lanes 4 and 10). The specificity of binding was also confirmed by using an 18 bp dsDNA oligonucleotide bearing the STRE1 motif and its boundary genomic sequences (Table S1) and a DNA probe containing the mutated motif STRE1 (Freitas and Bertolini 2004). The competitor dsDNA oligonucleotide abolished the DNA band shifts observed with the probes STRE1 and STRE2 (Figure 5B, lanes 5 and 11). In addition, the DNA-protein complex was not observed when using the mutated probe STRE1 (Figure 5B, lanes 14 and 15), thus confirming the specificity of the GST-SEB-1 binding to STRE1.

### The accumulation of reserve carbohydrates is regulated by SEB-1

**Figure 6:**
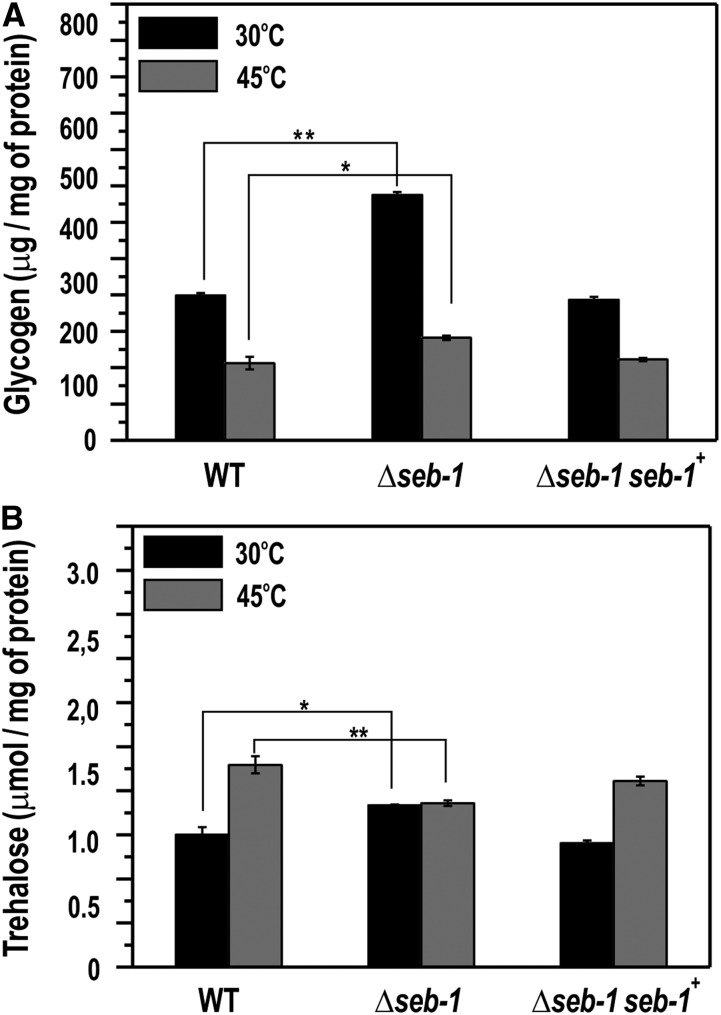
The Δ*seb-1* strain exhibits impaired accumulation of reserve carbohydrates during heat stress. Liquid cultures of the WT, Δ*seb-1*, and Δ*seb-1* complemented strains were exposed to a temperature shift from 30° to 45° for 30 min. After this, the heat-shocked mycelia were used to prepare a cellular extract used for glycogen and trehalose quantification, as described in the *Materials and Methods*. (A) Glycogen content. (B) Trehalose content. WT: wild-type strain; Δ*seb-1*: strain mutated in the ORF NCU02671; Δ*seb-1 seb-1*^+^: Δ*seb-1* complemented strain (Δ*seb-1 his-3*::*Pccg-1-seb1-sfgfp*). The error bars represent the standard deviation. Values of three biological replicates were used for statistical analysis and the significances (*P < 0.05, **P < 0.01) between the strains were estimated by the Tukey-Kramer multiple comparison test.

We described above that SEB-1 binds *in vitro* to the STRE motifs present in the *gsn* promoter and that the Δ*seb-1* strain was sensitive to heat, pH, oxidative, and osmotic stress. In order to correlate both results, we quantified the glycogen accumulated by the wild-type strain and the Δ*seb-1* and Δ*seb-1* complemented strains under normal growth temperature (30°) and after exposure to heat stress (transferring from 30° to 45°). In *N. crassa*, glycogen is accumulated at the end of the exponential growth phase and is degraded after heat shock (de Paula *et al.* 2002; Freitas and Bertolini 2004). The Δ*seb-1* strain exhibited the same profile of glycogen accumulation as compared to the wild-type strain, *i.e.*, lower levels after heat stress (Figure 6A). However, the mutant strain accumulated much higher glycogen levels than the wild-type strain under normal growth temperature, *i.e.*, before heat shock, indicating that SEB-1 affects glycogen accumulation under normal growth temperature (Figure 6A). SEB-1 also controls glycogen accumulation under heat stress, however, the major control seems to be at 30°.

We also quantified trehalose, another reserve carbohydrate. Trehalose is a nonreducing disaccharide found at high levels in fungi, which accumulates preferentially in spores to be used as a carbon and energy source for conidial germination (Hanks and Sussman 1969; Fillinger *et al.* 2001). In *N. crassa* conidia, trehalose corresponds to 10% of the dry-weight, and its levels decrease upon germination and remain at low levels during vegetative growth. Trehalose levels rise again at the end of the growth and accumulate in conidia (Hanks and Sussman 1969). A temperature-shift (from 30° to 45°) results in an accumulation of trehalose concomitant with a decrease in glycogen levels (Neves *et al.* 1991). These effects may depend on the activities of glycogen synthase, glycogen phosphorylase, and trehalose-phosphate synthase, the regulatory enzymes in glycogen and trehalose metabolism, respectively (Noventa-Jordão *et al.* 1996). SEB-1 affects trehalose levels under normal growth temperature and under heat stress. The levels were significantly reduced in the Δ*seb-1* under heat stress, while the levels under normal growth temperature showed a slight but significant increase (Figure 6B). The rescued strain showed similar glycogen and trehalose levels to the wild-type strain. All together, these findings suggest that SEB-1 influences the metabolism of both reserve carbohydrates in *N. crassa*.

### SEB-1 modulates the expression of all glycogenic genes and binds in vivo to their promoters

**Figure 7:**
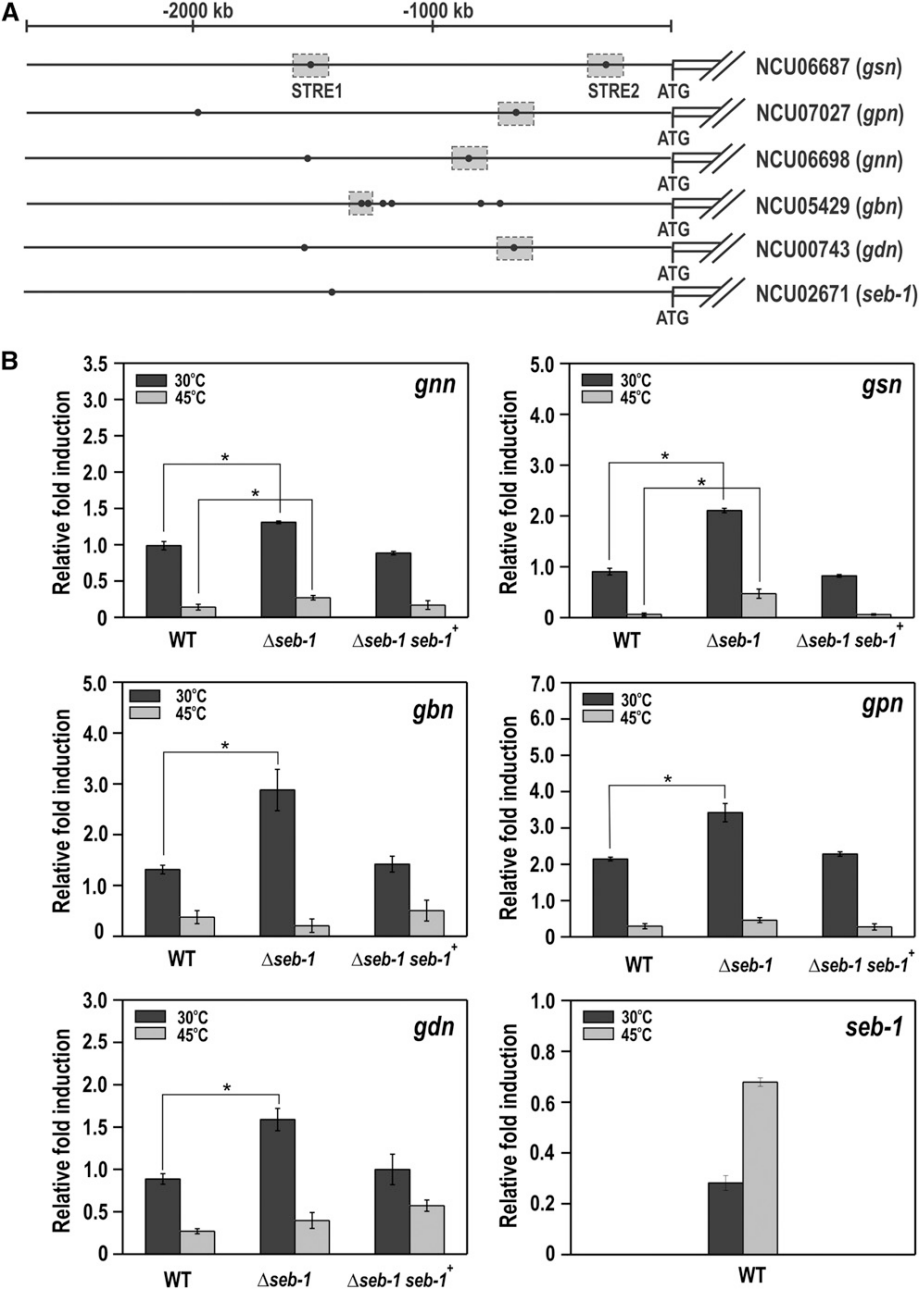
The expression of glycogenic genes is regulated by SEB-1. To analyze gene expression, mycelia were grown at 30° for 24 hr in VM (Vogel’s minimal) medium containing 2% sucrose, collected, and subjected to RNA extraction and cDNA synthesis. (A) Schematic representation of the genes including their 5′-flanking regions with the putative STRE motifs (filled black circles). Gray rectangles indicate the STRE motifs analyzed by ChIP-qPCR. (B) Gene expression analysis of the genes encoding glycogenic enzymes – glycogen synthase *gsn* (NCU06687), glycogen phosphorylase *gpn* (NCU07027), glycogenin *gnn* (NCU06698), glycogen branching enzyme *gbn* (NCU05429), glycogen debranching enzyme *gdn* (NCU00743) genes, and the *seb-1* gene (NCU02671). WT: wild-type strain; *seb-1*: strain mutated in the ORF NCU02671; Δ*seb-1 seb-1*^+^: Δ*seb-1* complemented strain (Δ*seb-1 his-3*::*Pccg-1-seb1-sfgfp*). Expression of the *act* gene (NCU04173) was used as the reference, and the wild-type samples at 30° and 45° were used as references samples at 30° and 45°, respectively. The error bars represent the standard deviation for each condition. Values of six replicates were used for statistical analysis and the significance (*P < 0.001) between strains was estimated by the Tukey-Kramer multiple comparison test. STRE, stress response element.

To investigate whether SEB-1 controls glycogen accumulation by regulating the genes encoding the glycogenic enzymes, we performed RT-qPCR on genes encoding enzymes of glycogen synthesis (*gnn*, *gsn*, and *gbn*) and glycogen degradation (*gpn* and *gdn*) in samples taken before (30°) and after heat shock (45°). An *in silico* analysis of the gene promoters revealed the existence of the STRE motif in all promoters, either adjacent to each other or separate (Figure 7A). A STRE motif was also identified in the *seb-1* promoter itself. All genes were overexpressed in the *seb-1* strain at normal growth temperature (30°, *P* < 0.001), indicating that SEB-1 acts as a repressor of the glycogenic genes under this condition (Figure 7B). It is important to note that the genes encoding enzymes for glycogen synthesis and degradation were highly expressed at 30°, which means that both processes would be similarly repressed by the transcription factor in the wild-type strain. However, the glycogen levels were higher in the *seb-1* strain under the same growth condition (see Figure 6A). Considering these results, we suggest that glycogen accumulation likely results from gene expression regulation and the balance between the enzyme activities of synthesis and degradation. It is important to emphasize that glycogen synthase and glycogen phosphorylase are enzymes regulated by phosphorylation. We also observed that the *gnn* and *gsn* genes (encoding enzymes of glycogen synthesis) were significantly overexpressed at 45° in the *seb-1* strain (*P* < 0.001, Figure 7B), which may explain the glycogen accumulated by the *seb-1* strain under this condition (see Figure 6A). Heat stress also induced the expression of the *seb-1* gene (Figure 7B) in the wild-type strain, a finding that is consistent with the involvement of this transcription factor in heat stress response. In this case, heat stress induced *seb-1* expression, whose product is required for the expression regulation of genes under stress.

**Figure 8:**
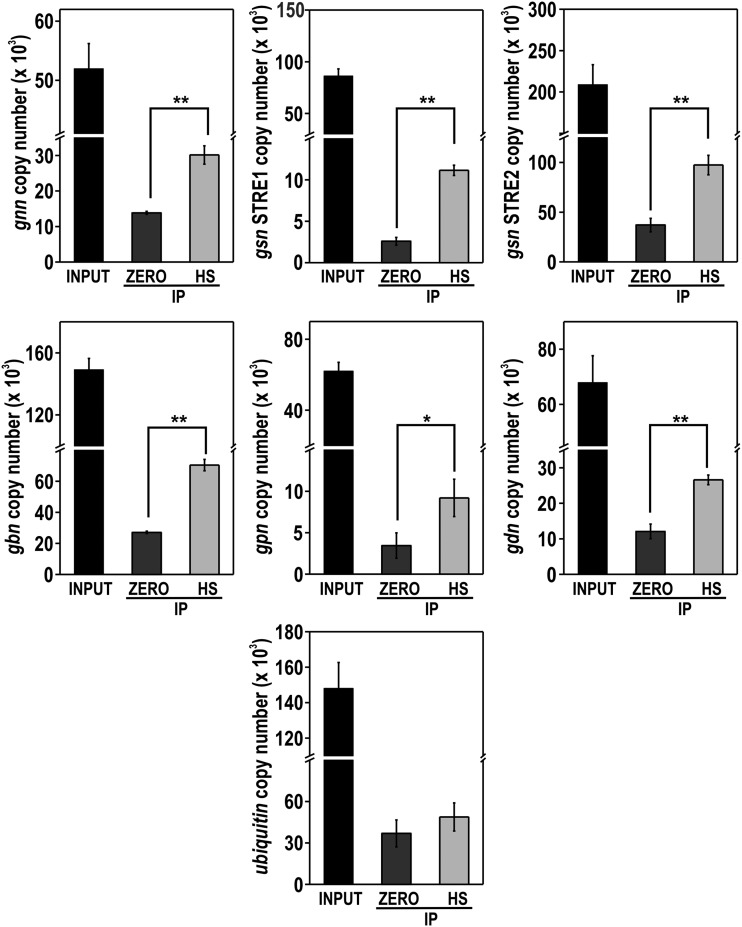
The transcription factor SEB-1 binds *in vivo* to the promoters of glycogenic genes. Genomic DNA from the Δ*seb-1* complemented strain (Δ*seb-1 his-3*::*Pccg-1-seb1-sfgfp*) subjected or not to heat stress was immunoprecipitated with anti-GFP antibody and the immunoprecipitates (IPs) were used to analyze the binding of SEB-1 to the target genes *gnn*, *gsn*, *gbn*, *gpn*, and *gdn* by ChIP-qPCR. A region inside the coding sequence of the ubiquitin gene was used as a negative control for binding. Values of three replicates were used for statistical analysis (*P < 0.05, **P < 0.01, estimated by the Tukey-Kramer multiple comparison test) and the error bars represent the standard deviation for each condition. INPUT: genomic DNA before IP; ZERO: mycelium from a culture at 30° for 24 hr, HS: mycelium from a culture submitted to heat stress (45°) for 30 min; IP: genomic DNA after anti-GFP immunoprecipitation.

ChIP-qPCR assays were performed to confirm the regulation of all the genes by SEB-1. All genes involved in glycogen metabolism have STRE motifs in their 5′-flanking regions and some of them were analyzed for *in vivo* binding (the gray boxes in Figure 7A). In these experiments, we used the Δ*seb-1* complemented strain (*his-3*::*Pccg-1-seb-1-sfgfp*) and anti-GFP antibody. Chromatin was collected from mycelia before (sample zero) and after heat stress (sample HS) and binding of SEB-1 was quantified in the *gsn*, *gpn*, *gdn*, *gnn*, and *gbn* promoters. The input DNA was used as a positive control and a fragment of the ubiquitin gene lacking the motif was used as a negative control for binding. SEB-1 bound to all motifs analyzed both before and after heat stress; however, binding was significantly increased under heat stress (Figure 8). It is noteworthy to observe the high amplification of the *gsn* gene when using the STRE2 probe, which may suggest a major role for this motif in the regulation of *gsn* expression by SEB-1 (compare with the amplification of the STRE1 probe). Together, these results indicated that SEB-1 recognized and bound to all glycogenic gene promoters *in vivo*, before and after heat shock; such binding would control the glycogen levels under both conditions through the regulation of gene expression.

### Determining the SEB-1 regulon

**Figure 9:**
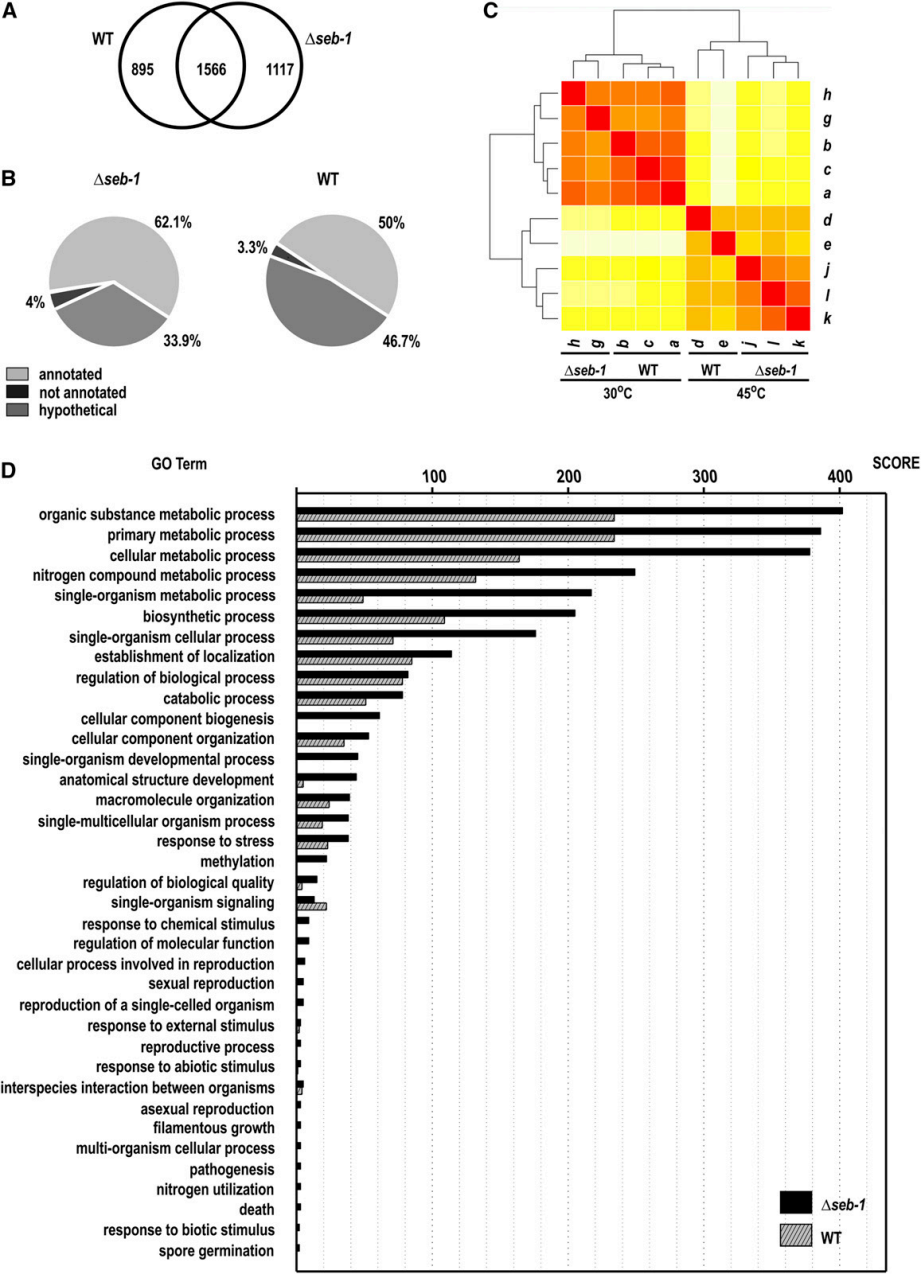
The *seb-1* regulon. (A) Venn diagram showing the overlap between the differentially expressed genes exhibiting a statistically significant profile during heat shock in the wild-type and Δ*seb-1* strains (*P* < 0.05). (B) Distribution of the ORFs (open reading frames) identified by RNA-seq. (C) Heat map showing Euclidean distances between wild-type (WT) and Δ*seb-1* strains under heat shock. The darker the color, the closer are the two datasets. a, b, and c, RNA-seq libraries from the wild-type strain at 30°; d and e, RNA-seq libraries from the wild-type strain at 45°; g and h, RNA-seq libraries from the Δ*seb-1* strain at 30°; j, k, and l, RNA-seq libraries from the Δ*seb-1* strain at 45°. (D) Distribution of differentially expressed genes showing a statistically significant profile in the response to shock in the wild-type and Δ*seb-1* strains, according to functional categories (GO terms). The scores represent the number of ORFs identified as differentially expressed. GO terms are listed on the left and the Blast2GO score of molecular function is shown on top. Gene Ontology (GO) analyses were performed using the Blast2GO web tool, as described in *Material and Methods*.

In *S. cerevisiae*, the *cis* regulatory motif STRE is involved in the response to different stress conditions such as heat shock, osmotic stress, and oxidative stress, and requires the Msn2/4p proteins as transactivators (Martínez-Pastor *et al.* 1996). In *T. atroviride*, the transcription factor Seb1 was also described as binding to STRE in response to osmotic stress (Seidl *et al.* 2004) and the *A. fumigatus* SebA protein is involved in the responses to heat and oxidative stress, nutrient starvation, and pathogenesis (Dinamarco *et al.* 2012). In this work, we showed that the *N. crassa* Seb-1/SebA ortholog is the SEB-1 protein, a transcription factor involved in the response to a variety of stresses, as demonstrated above. We characterized the SEB-1 regulon by RNA-seq by analyzing the genome-wide transcriptional profile of the Δ*seb-1* strain during vegetative growth (30°) and after shifting it to a high temperature (45°). As a control, we performed the same analysis with the wild-type strain grown at both temperatures. When we compared the Δ*seb-1* strain before and after heat stress, the number of transcripts identified after heat stress was high. A total of 2683 ORFs (28.4% of the annotated genome) showed an expression profile that was significantly affected by the *seb-1* deletion (adjusted to *P* < 0.05). Of these, 1117 ORFs were significantly dependent on the functional SEB-1 transcription factor (Figure 9A), indicating that these were genes likely regulated directly or indirectly by SEB-1 under heat stress. Of the genes regulated by SEB-1 under heat stress, 62.1% (693 ORFs) encode proteins annotated in public databases, 33.9% (379 ORFs) encode proteins classified as hypothetical proteins, and 4% (45 ORFs) encode sequences not yet annotated (Figure 9B and Table S2). The overall similarity among each of the samples was calculated using the Euclidean distance and transcript abundance values for all genes and data sets were clustered (Figure 9C). These analyses indicated that the data sets generated from mycelia exposed to heat stress were significantly different from those of mycelia not exposed to heat stress. Additionally, the samples from the *seb-1* strain exposed to heat stress (j, k, and l) formed a separate clade from those of the wild-type strain under the same condition (d and e). This event was also observed before heat shock, *i.e.*, there were separate clades between the wild-type and *seb-1* strains during vegetative growth (30°), indicating that under this condition there were genes whose expression was modulated by SEB-1.

An analysis of the 1117-gene set identified as differentially regulated in the Δ*seb-1 vs.* wild-type strains (Table S2) was used to predict the function of each gene based on the Gene Ontology (GO) categories using Blast2GO (Conesa *et al.* 2005). The most represented GO categories within the differentially expressed genes under heat stress were identified using Fisher’s exact test, with an adjusted *P* < 0.05 (Table S3 and Table S4). The 1117 genes and their respective GO terms are represented in Figure 9D. Among the highly enriched biological processes, genes related to general stress responses that include cellular response to osmotic stress, starvation, autophagy, lipid modifications, response to oxidative stress, and response to chemical stimulus were identified (Table S3). Many GO terms enriched only in the *seb-1* strain data set as compared with the wild-type strain, such as cellular component biogenesis, single-organism developmental processes, and methylation, while other categories, such as those belonging to metabolic processes, were highly represented in the Δ*seb-1* strain compared to the wild-type strain (Figure 9D).

**Figure 10:**
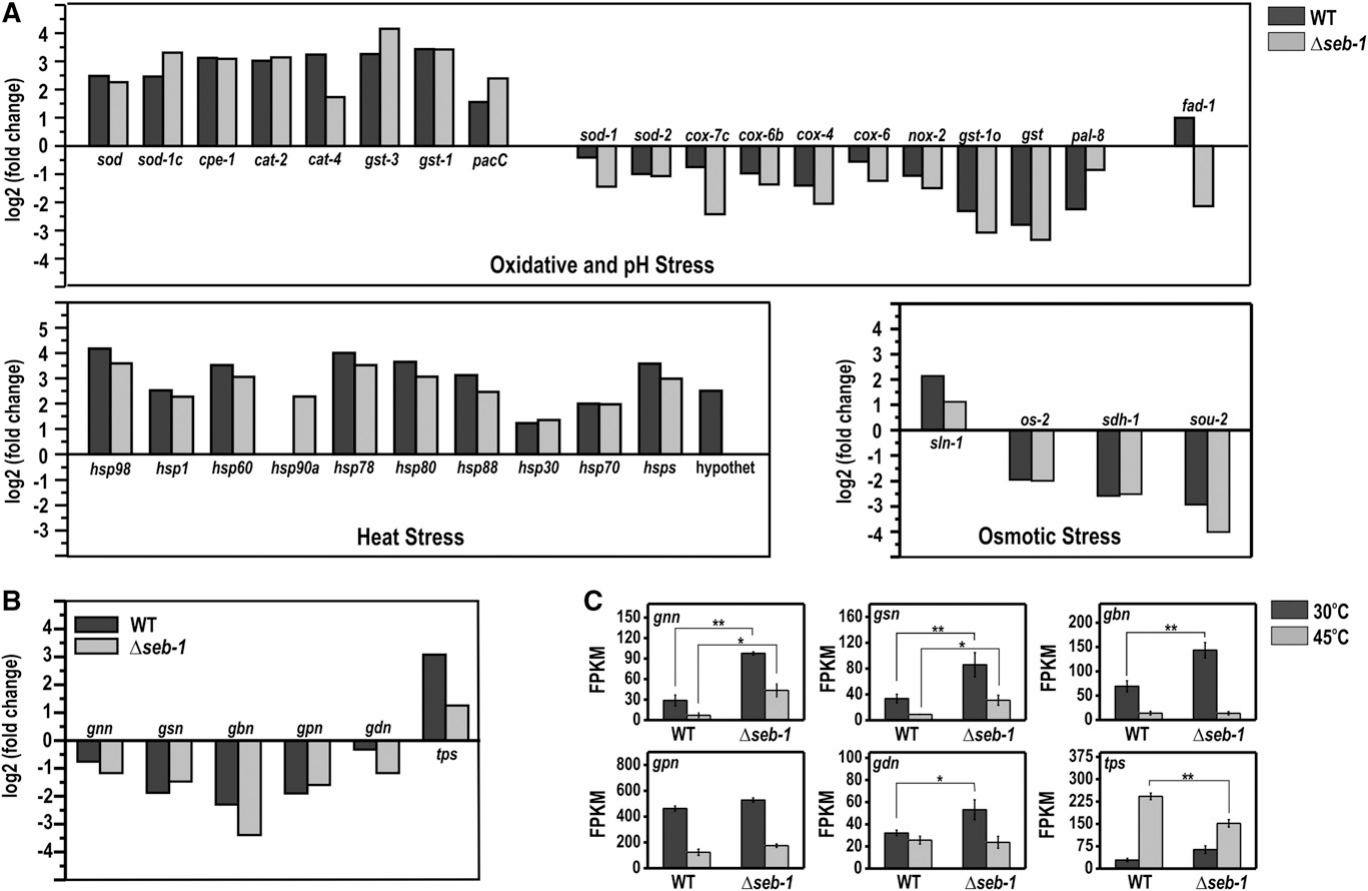
SEB-1 controls the expression of genes involved in glycogen and trehalose metabolism and some stress responsive genes under heat stress. The expression levels were indicated as fold change (log2) and FPKMs (fragments per kb of transcript per million mapped reads). (A) Genes involved in oxidative, pH, heat, and osmotic stress responses in wild-type (WT) and Δ*seb-1* strains exposed to heat stress (45°). (B) Genes involved in glycogen and trehalose metabolism in WT and Δ*seb-1* strains exposed to heat stress (45°). (C) Genes involved in glycogen and trehalose metabolism in the WT and Δ*seb-1* strains exposed or not exposed to heat stress (45°). *gnn*, glycogenin (NCU06698); *gsn*, glycogen synthase gene (NCU06687); *gbn*, glycogen branching enzyme gene (NCU05429); *gpn*, glycogen phosphorylase gene (NCU07027); *gdn*, glycogen debranching enzyme gene (NCU00743); *sod*, superoxide dismutase variant gene (NCU09560); *sod-1*, superoxide dismutase-1 gene (NCU02133); *sod-2*, superoxide dismutase-2 gene (NCU01213); *cat-2*, catalase-2 gene (NCU05770); *cat-4*, catalase-4 gene (NCU05169); *os-2*, osmotic sensitive-2 gene (NCU07024); *pacC*, pH-response transcription factor pacC/RIM101 gene (NCU00090). The error bars represent the standard deviation for each condition, with triplicate measurements. Values of three replicates were used for statistical analysis and the significances (*P < 0.01 and **P < 0.001) between strains were estimated by the Tukey-Kramer multiple comparison test.

Many of the 1117 genes that comprise the *seb-1* regulon have an annotated function within the stress response category. For the oxidative stress response pathway, four cytochrome c oxidases (*cox-7c*, NCU03340; *cox-4*, NCU05689; *cox-6*, NCU06695; and *cox-6b*, NCU06741), a cytochrome c peroxidase (*cpe-1*, NCU03297), a NADPH-oxidase (*nox-2*, NCU10775), a FAD-dependent sulfhydryl oxidase (*fad-1*, NCU09291), four superoxide dismutases (*sod-2*, NCU01213; *sod-1*, NCU02133; *sod-1c*, NCU07851; and *sod*, NCU09560), two catalases (*cat-4*, NCU05169 and *cat-2*, NCU05770), and four glutathione transferases (*gst-1o*, NCU00549; *gst-3*, NCU01320; *gst*, NCU04676; and *gst-1*, NCU05780) were identified (Figure 10A, see oxidative and pH stress panel). Heat stress also regulated the expression of many genes previously described as genes belonging to the pH-stress response pathway, such as the pH-responsive *pacC* gene (NCU00090) (Cupertino *et al.* 2012) and the *A. nidulans palH* orthologous gene (termed *pal-8*, NCU00007) (Figure 10A, see oxidative and pH stress panel). For the osmotic stress pathway, heat stress induced *sln-1* (osmolarity two-component system protein, NCU04615) and repressed *os-2* (osmotic sensitive-2, NCU07024), *sdh-1* (sorbitol dehydrogenase 1, NCU01905), and *sou-2* (sorbitol utilization protein 2, NCU03803) genes (Figure 10A, see osmotic stress panel). As expected, all genes identified by RNA-seq (*P* < 0.05) within heat stress response, such as those encoding heat shock proteins (*hsp98*, NCU00104; *hspSTI1*, NCU00714; *hsp60*, NCU01589; *hsp90a*, NCU01792; *hsp78*, NCU02630; *hsp80*, NCU04142; *hsp88*, NCU05269; *hsp30*, NCU07232; *hsp70*, NCU08693; and *hsps*, NCU09602) and a hypothetical protein (NCU01788), were induced after shifting the mycelia from 30° to 45° (Figure 10A, see heat stress panel). Expression of the *hsp90a* gene and the gene encoding a hypothetical protein was SEB-1-dependent.

All of the genes encoding glycogen metabolism enzymes, *i.e.*, glycogenin (*gnn*, NCU06698), glycogen synthase (*gsn*, NCU06687), glycogen branching enzyme (*gbn*, NCU05429), glycogen phosphorylase (*gpn*, NCU07027), and glycogen debranching enzyme (*gdn*, NCU00743) were repressed by heat stress (Figure 10B, see FPKM graphs), consistent with the results shown in Figure 7B. The effect of the *seb-1* deletion was clearly demonstrated for these genes before heat stress, since all of them showed expression levels significantly higher than those observed in the wild-type strain during vegetative growth (30°). These results suggest a repressor role for SEB-1 on the expression of these genes under this environmental condition. In addition, these results also suggested that the main targets of regulation are genes involved in glycogen synthesis (Figure 10C, *gnn*, *gsn*, and *gbn*, FPKM panels). This finding is very important since it is consistent with the higher glycogen levels observed in the Δ*seb-1* strain compared to the wild-type strain (see Figure 6A). On the other hand, the gene encoding trehalose phosphate synthase (*tps*, NCU00793), the rate-limiting enzyme in trehalose synthesis, was induced after heat stress in both strains (Figure 10C, see *tsp* FPKM graph). However, the expression of this gene was lower in the mutant strain under heat stress. These results were consistent with the trehalose levels in the wild-type strain and could explain the levels observed in the Δ*seb-1* strain under heat stress (see Figure 6B). The results presented here support our hypothesis that the SEB-1 transcription factor plays a role as an activator and/or a repressor for different genes, depending on the environmental condition.

## Discussion

All living cells have the ability to respond and adapt to environmental changes by using cellular mechanisms that integrate environmental sensing and signal transduction pathways, which lead to the induction or repression of gene expression. The availability of whole genome sequences allows comparison of the global responses to diverse environmental stresses in different organisms. Comparative studies have revealed the existence of conserved mechanisms in addition to specific responses that highlight unique defense systems (Gasch *et al.* 2000; Swan and Sistonen 2015). Genetic and cell biological studies using different organisms have led to the discovery of global stress regulators and mechanisms conferring resistance to adverse environmental conditions. As a result, knowledge of the molecular mechanisms of adaptation to stress conditions in fungi is extensive (Estruch 2000; Gasch *et al.* 2000; Duran *et al.* 2010; Berry *et al.* 2011; Kroll *et al.* 2014; Brown *et al.* 2014; and others). In this work, we developed an approach that included capturing proteins using streptavidin-conjugated beads/biotinylated STRE oligonucleotides followed by mass spectrometry to identify *N. crassa* protein(s) able to bind to STRE, a DNA element present in the upstream regions of stress responsive genes in *S. cerevisiae*. Among the candidate proteins identified was SEB-1. Although orthologs of this protein have been reported to be involved in stress responses in a few filamentous fungi (Peterbauer *et al.* 2002; Dinamarco *et al.* 2012), its requirement for resistance to different stress responses may vary among organisms. Whereas the *T. atroviride* Seb1 transcription factor is only involved in osmotic stress (Peterbauer *et al.* 2002), the *A. fumigatus* SebA appears to play no role in osmotic stress, and instead is required for the response to heat and oxidative stresses and poor nutrient conditions (Dinamarco *et al.* 2012). Our data indicates that the *N. crassa* SEB-1 transcription factor is involved in the response to heat, pH, osmotic, and oxidative stresses. These data suggest that SEB-1 orthologs in different fungi may function by targeting different signaling pathways, resulting in differences in cellular responses to stress that are dependent upon SEB-1 orthologs.

We identified SEB-1 as the *N. crassa* transcription factor capable of binding to the STRE sequence (AGGGG), which is the nucleotide sequence recognized by the Msn2/4p transcription factors in *S. cerevisiae*. The ability of SEB-1 to bind to the STRE sequence was further confirmed by showing that SEB-1 binds *in vitro* to STRE sequences present in the promoter of the gene encoding an enzyme of glycogen metabolism (*gsn*). SEB-1 regulates the expression of all glycogenic genes (see Figure 7 and Figure 10B) and the *tps* gene, which encodes trehalose phosphate synthase, the enzyme that catalyzes the first step in trehalose synthesis, consistent with a role for SEB-1 in glycogen and trehalose metabolism. However, gene expression was either up- or down-regulated in the Δ*seb-1* strain (see Figure 10B). This double regulatory function of SEB-1 was also observed for the expression of genes involved in oxidative, pH, heat, and osmotic stress. Since all glycogenic genes have STRE in their promoter regions and, in yeast, STRE mediates the activation of stress-responsive gene, these data suggest that either STRE is not required for the gene expression regulation by SEB-1 or that SEB-1 requires interaction with additional transcription factors and/or proteins in *N. crassa*. The *A. fumigatus* SebA transcription factor has also been described as regulating gene expression by activating or repressing genes involved in the heat stress response (Dinamarco *et al.* 2012). All these data reinforce the hypothesis that the transcription factors of the SEB family are not the real yeast Msn2/4p functional homologs; rather, they are proteins that regulate stress responses likely involving an alternative mechanism of action.

In yeast, trehalose-6-P synthase (Tps1), but not trehalose, is indispensable for withstanding high temperature and oxidative stresses. It was suggested that Tps1 is a sensing/signaling intermediate with regulatory function(s), at least regarding energy homeostasis (Petitjean *et al.* 2015; Gibney *et al.* 2015). As shown here, SEB-1 regulated the expression of genes related to reserve carbohydrate metabolism and also genes often induced by stress, which includes the *hsp*, *cat*, and *sod* genes, among others. In the latter case, the majority of genes were up-regulated by SEB-1 (see Figure 8A). Whether the genes involved in reserve carbohydrate metabolism are regulated by SEB-1 in the same manner as stress-responsive genes, or whether the regulation of such genes is required for stress survival (as shown here), deserves further investigation. In *S. cerevisiae*, some genes involved in central carbon metabolism, such as *PYC1* (pyruvate carboxylase), *PFK1* (phosphofructokinase), and *CIT2* (citrate synthase), were described as having a fundamental role in heat-shock resistance. In addition, genes involved in cellular signaling and chromatin regulation were also related to confer hypersensitivity to heat, suggesting a connection between heat shock and these processes (Gibney *et al.* 2013).

We used RNA-seq to determine the expression profile of the Δ*seb-1* strain under heat stress (by transferring from 30° to 45°). Using this approach, we demonstrated that SEB-1 might connect the heat stress response to a broad range of cellular mechanisms, including chromatin architecture, as would be expected under this environmental condition. We observed a high number of genes implicated in this process as a putative target of regulation by SEB-1. All genes encoding histone proteins were differently expressed in the Δ*seb-1* strain, being down-regulated under heat stress. In addition, genes encoding proteins involved in histone modifications, such as the histone deacetylases HAD-2 and HAD-3 (Smith *et al.* 2010) and the histone acetyltransferase HAT-4 (Borkovich *et al.* 2004), were overexpressed in the Δ*seb-1* strain. Interestingly, we also identified the ORFs NCU03482 and NCU06679 as genes differently regulated by SEB-1 under heat stress; the proteins encoded by these genes were previously described in *N. crassa* as STRE-binding proteins (Freitas *et al.* 2008). The NCU06679 gene, previously annotated as *cac-3* (chromatin assembly complex 3, Borkovich *et al.* 2004), was recently named as *npf*, a component of a multimeric complex involved in histone methylation in *Neurospora* (Jamieson *et al.* 2013), whereas NCU03482 is a DNA helicase homologous to the mammalian RuvBL1 protein. This latter protein, together with RuvBL2, belongs to the AAA+ (ATPases associated with various cellular activities) protein family and is highly conserved in eukaryotes. They are known by multiple names, depending on the organism, and are components of several multiprotein complexes involved in a wide range of cellular processes, including chromatin remodeling and transcriptional regulation (Nano and Houry 2013).

The transcriptomic results also provided evidence that SEB-1 controls the expression of genes encoding protein components of the circadian clock in *N. crassa*. Genes encoding the proteins WC-1 (white collar-1), VIVID, and the Frequency-Interacting RNA Helicase protein (FRH) were clearly up-regulated in the Δ*seb-1* strain under heat stress, suggesting that the clock mechanism may be impaired in the mutant strain under such conditions. The clock-controlled gene-8 (*ccg-8*) was also identified as regulated by SEB-1. Since the mutant strain also exhibited impairment in the accumulation of the reserve carbohydrate glycogen and trehalose under heat stress, it is tempting to suggest that SEB-1 could play a role in coordinating the carbohydrate metabolism controlled by the clock in *N. crassa*. The connection between the circadian clock and metabolism has been under investigation in recent years (Asher and Sassone-Corsi 2015). Many genes involved in metabolism have recently been identified as clock-controlled genes in a *N. crassa* transcriptomic assay, including some genes involved in trehalose metabolism (Hurley *et al.* 2014). The results of this analysis revealed that much of metabolism is clock-controlled; daytime favors catabolism and nighttime favors the biosynthesis of cellular components. Regarding the clock-control of glycogen metabolism, Doi *et al.* (2010) have shown that the mouse CLOCK transcription factor regulates the circadian rhythms of hepatic glycogen synthesis through transcriptional activation of Gys2, the gene encoding glycogen synthase. In *N. crassa*, the *gsn* and *gpn* genes, which encode glycogen synthase and the glycogen phosphorylase enzymes, respectively, have been reported to be light-regulated genes (Wu *et al.* 2014).

Autophagy is another cellular process that may be regulated by SEB-1, since the *ATG8* gene is a target of regulation under heat stress. A number of genes have been implicated in this process and the Atg8 protein was described as a key component of the autophagosome in yeast (Xie *et al.* 2008). Interestingly, Wang *et al.* (2001) reported a connection between glycogen metabolism and autophagy mediated by the action of the protein kinases Snf1p and Pho85p. We also identified the SNF-1 protein kinase as a target of regulation by SEB-1. A high number of proteins implicated in a variety of additional cellular processes were also identified as targets of regulation by SEB-1, including membrane transporters, proteins involved in carbohydrate, nitrogen, and calcium metabolism, and light-responsive proteins. However, the regulation of these processes by SEB-1 and the conditions in which the processes are regulated in *N. crassa* require further investigation. Taken together, all our data suggest that SEB-1 acts as a regulatory hub in a network linking different cellular processes.

## Supplementary Material

Supplemental Material
