# Supplementary material for: The SEB-1 Transcription Factor Binds to the STRE Motif in Neurospora crassa and Regulates a Variety of Cellular Processes Including the Stress Response and Reserve Carbohydrate Metabolism
Source: G3 (Bethesda). 2016 Mar 16;6(5):1327–43. doi: 10.1534/g3.116.028506 (PMC4856084; doi:10.1534/g3.116.028506)
Supplement: Supplemental Material [file supp_6_5_1327__index.html]

The SEB-1 Transcription Factor Binds to the STRE Motif in Neurospora crassa and Regulates a Variety of Cellular Processes Including the Stress Response and Reserve Carbohydrate Metabolism — The SEB-1 Transcription Factor Binds to the STRE Motif in Neurospora crassa and Regulates a Variety of Cellular Processes Including the Stress Response and Reserve Carbohydrate Metabolism — Supplemental Material 

# The SEB-1 Transcription Factor Binds to the STRE Motif in *Neurospora crassa* and Regulates a Variety of Cellular Processes Including the Stress Response and Reserve Carbohydrate Metabolism

## Supplemental Material for Freitas *et al.*, 2016

**Files in this Data Supplement:**

- File S1 - Supplemental Materials and Methods. File also contains legends for Figures S1-S3 and Tables S1-S4. (.pdf, 175 KB)
- Figure S1 - The Δ*seb-1* strain is sensitive to pH stress. (.pdf, 176 KB)
- Figure S2 - The Δ*seb-1* strain is sensitive to oxidative stress. (.pdf, 426 KB)
- Figure S3 - The Δ*seb-1* strain is sensitive to high osmolarity. (.pdf, 353 KB)
- Table S1 - Oligonucleotides used in this work. (.pdf, 219 KB)
- Table S2 - Genes differentially expressed under heat stress identified by RNA-seq. (.xlsx, 2,032 KB)
- Table S3 - GO categories of the differentially expressed genes. (.xlsx, 34 KB)
- Table S4 - Enrichment of genes necessary for heat tolerance by Fisher test. (.xlsx, 16 KB)
