# Supplementary material for: The SEB-1 Transcription Factor Binds to the STRE Motif in Neurospora crassa and Regulates a Variety of Cellular Processes Including the Stress Response and Reserve Carbohydrate Metabolism
Source: G3 (Bethesda). 2016 Mar 16;6(5):1327–43. doi: 10.1534/g3.116.028506 (PMC4856084; doi:10.1534/g3.116.028506)
Supplement: Supplemental Material [file supp_g3.116.028506_FileS1.pdf]

File S1. SUPPORTING INFORMATION

Materials and Methods

Phenotypic analysis and stress sensitivity

The effect of osmotic stress was observed by cultivating conidia from each strain at 30ºC in solid and liquid VM medium and increasing quantities of the stressing agents, either sorbitol or NaCl at concentrations ranging from 100 mM to 1.5 M. For oxidative stress, conidia from each strain were cultivated at 30ºC on solid and liquid VM containing increasing amounts of either paraquat or menadione or hydrogen peroxide, at concentrations ranging from 12.5 to 500 μM, 10 to 500 μM, and 1 to 20 mM, respectively. Alkaline and acid pH stress was also evaluated by cultivating the strains conidia on plates containing solid VM medium at pH 7.8 and 4.2, respectively, at 30ºC.

Figure Legends

Figure S1 The Δ*seb-1* strain is sensitive to pH stress. The sensitivity of the wild-type, Δ*seb-1*, and Δ*seb-1* complemented strains to pH was evaluated by cultivating the strains at 30ºC for two days at three different pH values: pH 5.8, 4.2, and 7.2. WT: wild-type strain; Δ*seb-1*: strain mutated in the ORF NCU02671; Δ*seb-1 seb-1*$^{+}$: Δ*seb-1* complemented strain (Δ*seb-1 his-3::Pccg-1-seb1-sfgfp*).

Figure S2 The Δ*seb-1* strain is sensitive to oxidative stress. The sensitivity of the wild-type, Δ*seb-1*, and Δ*seb-1* complemented strains to agents that induce oxidative

stress was evaluated in the presence of hydrogen peroxide, menadione, and paraquat. (A) The strains were inoculated on plates with increasing concentrations of hydrogen peroxide (1 to 20 mM), menadione (10 to 500 mM), and paraquat (25 to 500 mM), and cultivated at 30ºC for 24 and 48 h. (B) Growth of the strains was evaluated in liquid VM medium containing varying concentrations of the same agents. WT: wild-type strain; Δ*seb-1*: strain mutated in the ORF NCU02671; Δ*seb-1 seb-1*$^{+}$: Δ*seb-1* complemented strain (Δ*seb-1 his-3::Pccg-1-seb1-sfgfp*).

Figure S3 The Δ*seb-1* strain is sensitive to high osmolarity. (A) The wild-type, Δ*seb-1*, and Δ*seb-1* complemented strains were inoculated on solid VM medium containing increased concentrations of NaCl (0.1 to 1.5 M) and sorbitol (0.1 to 1.5 M) and cultivated at 30ºC for 24 and 48 h. (B) Growth of the strains was evaluated in liquid VM medium containing varying concentrations of NaCl and sorbitol. WT: wild-type strain; Δ*seb-1*: strain mutated in the ORF NCU02671; Δ*seb-1 seb-1*$^{+}$: Δ*seb-1* complemented strain (Δ*seb-1 his-3::Pccg-1-seb1-sfgfp*).

Table S1 Oligonucleotides used in this work

Table S2 Genes differentially expressed under heat stress identified by RNA-seq

Table S3 GO categories of the differentially expressed genes

Table S4 Enrichment of genes necessary for heat tolerance by Fisher test
