# Supplementary material for: The SEB-1 Transcription Factor Binds to the STRE Motif in Neurospora crassa and Regulates a Variety of Cellular Processes Including the Stress Response and Reserve Carbohydrate Metabolism
Source: G3 (Bethesda). 2016 Mar 16;6(5):1327–43. doi: 10.1534/g3.116.028506 (PMC4856084; doi:10.1534/g3.116.028506)
Supplement: Supplemental Material [file supp_g3.116.028506_FigureS1.pdf]

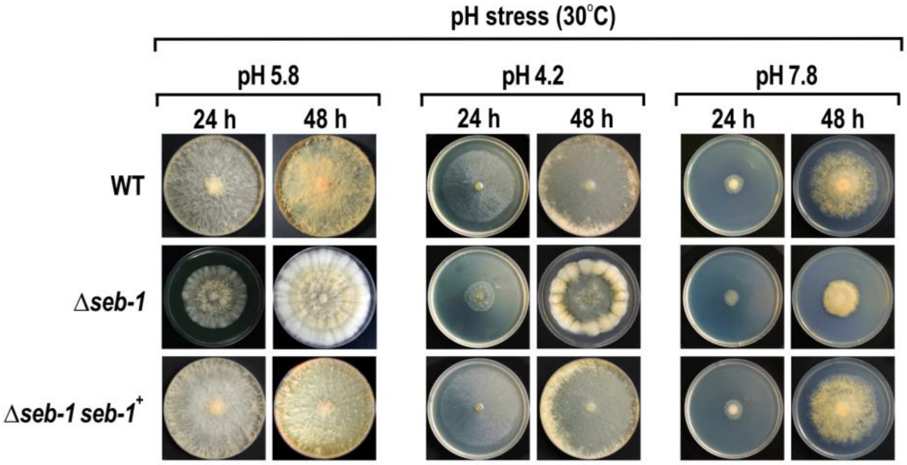

Figure S1. The Δ*seb-1* strain is sensitive to pH stress. The sensitivity of the wild-type, Δ*seb-1*, and Δ*seb-1* complemented strains to pH was evaluated by cultivating the strains at 30ºC for two days at three different pH values: pH 5.8, 4.2, and 7.2. WT: wild-type strain; Δ*seb-1*: strain mutated in the ORF NCU02671; Δ*seb-1 seb-1*$^{+}$: Δ*seb-1* complemented strain (Δ*seb-1 his-3::Pccg-1-seb1-sfgfp*).
