# Supplementary material for: The SEB-1 Transcription Factor Binds to the STRE Motif in Neurospora crassa and Regulates a Variety of Cellular Processes Including the Stress Response and Reserve Carbohydrate Metabolism
Source: G3 (Bethesda). 2016 Mar 16;6(5):1327–43. doi: 10.1534/g3.116.028506 (PMC4856084; doi:10.1534/g3.116.028506)
Supplement: Supplemental Material [file supp_g3.116.028506_FigureS2.pdf]

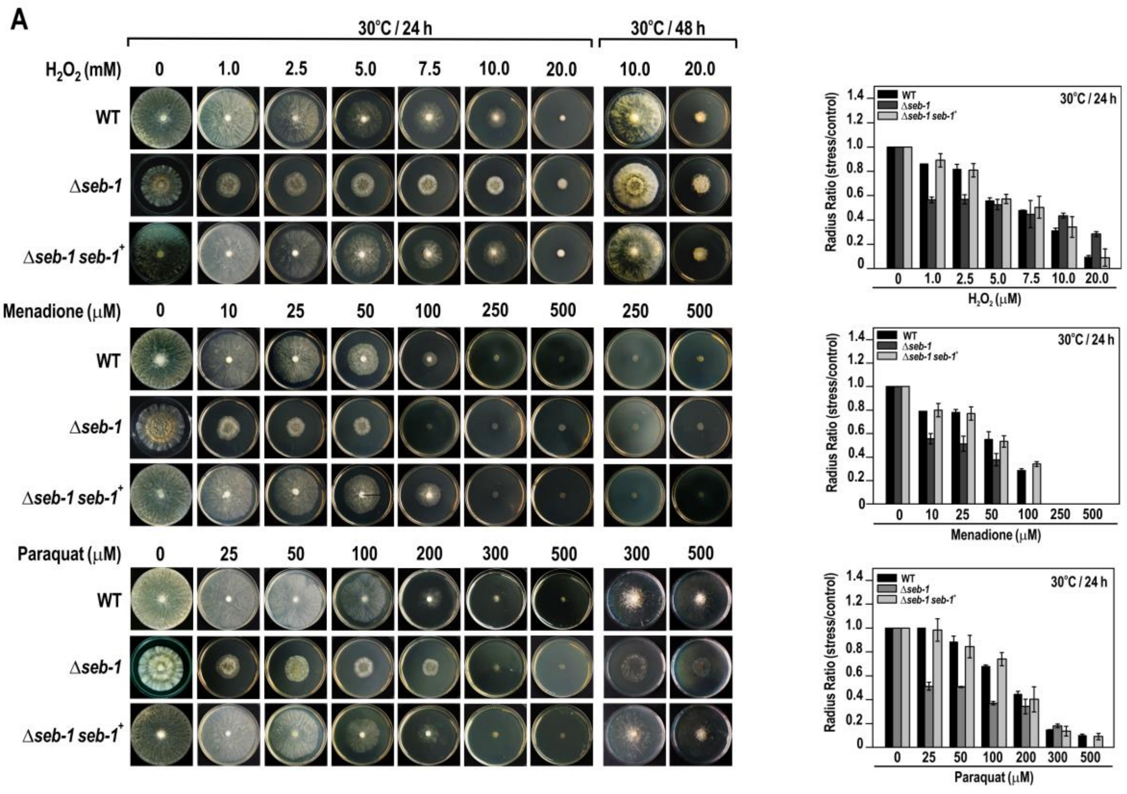
A
30°C / 24 h
30°C / 48 h
H2O2 (mM)
0 1.0 2.5 5.0 7.5 10.0 20.0 10.0 20.0
WT
Δseb-1
Δseb-1 seb-1+
Menadione (μM)
0 10 25 50 100 250 500 250 500
Paraquat (μM)
0 25 50 100 200 300 500 300 500
Radius Ratio (stress/control)
H2O2 (μM)
Menadione (μM)
Paraquat (μM)

Fig. S2 A

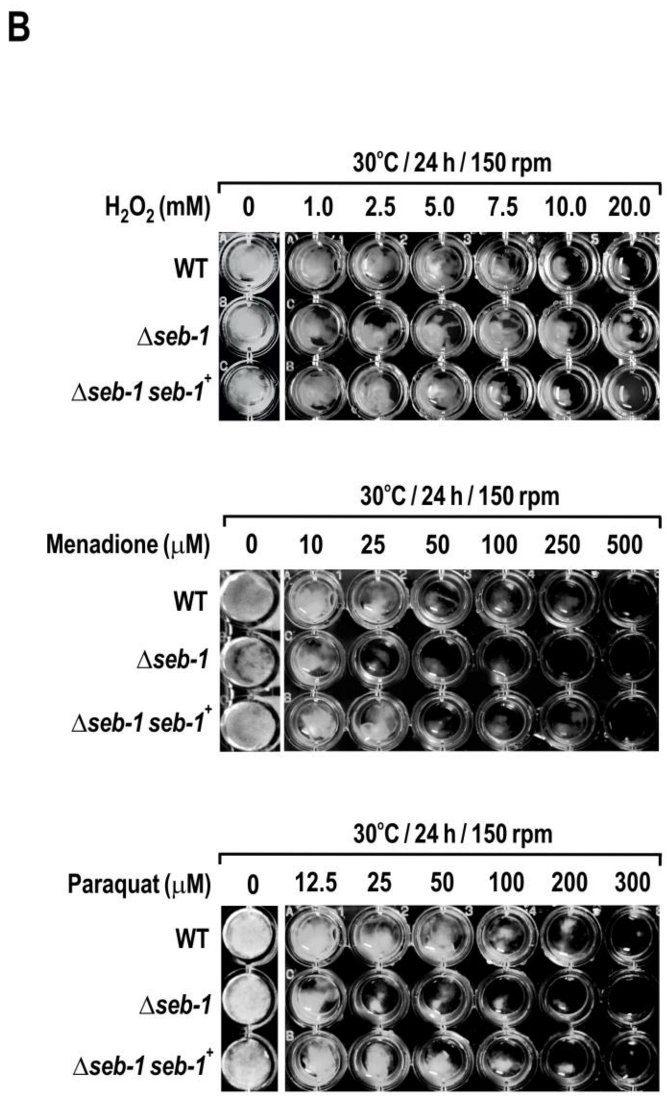
B
30°C / 24 h / 150 rpm
H2O2 (mM) 0 1.0 2.5 5.0 7.5 10.0 20.0
WT
Δseb-1
Δseb-1 seb-1+
30°C / 24 h / 150 rpm
Menadione (μM) 0 10 25 50 100 250 500
WT
Δseb-1
Δseb-1 seb-1+
30°C / 24 h / 150 rpm
Paraquat (μM) 0 12.5 25 50 100 200 300
WT
Δseb-1
Δseb-1 seb-1+

Fig. S2 B

Figure S2. The Δ*seb-1* strain is sensitive to oxidative stress. The sensitivity of the wild-type, Δ*seb-1*, and Δ*seb-1* complemented strains to agents that induce oxidative stress was evaluated in the presence of hydrogen peroxide, menadione, and paraquat. (A) The strains were inoculated on plates with increasing concentrations of hydrogen peroxide (1 to 20 mM), menadione (10 to 500 mM), and paraquat (25 to 500 mM), and cultivated at 30ºC for 24 and 48 h. (B) Growth of the strains was evaluated in liquid VM medium containing varying concentrations of the same agents. WT: wild-type strain; Δ*seb-1*: strain mutated in the ORF NCU02671; Δ*seb-1 seb-1*$^{+}$: Δ*seb-1* complemented strain (Δ*seb-1 his-3::Pccg-1-seb1-sfgfp*).
