# Supplementary material for: The SEB-1 Transcription Factor Binds to the STRE Motif in Neurospora crassa and Regulates a Variety of Cellular Processes Including the Stress Response and Reserve Carbohydrate Metabolism
Source: G3 (Bethesda). 2016 Mar 16;6(5):1327–43. doi: 10.1534/g3.116.028506 (PMC4856084; doi:10.1534/g3.116.028506)
Supplement: Supplemental Material [file supp_g3.116.028506_FigureS3.pdf]

A

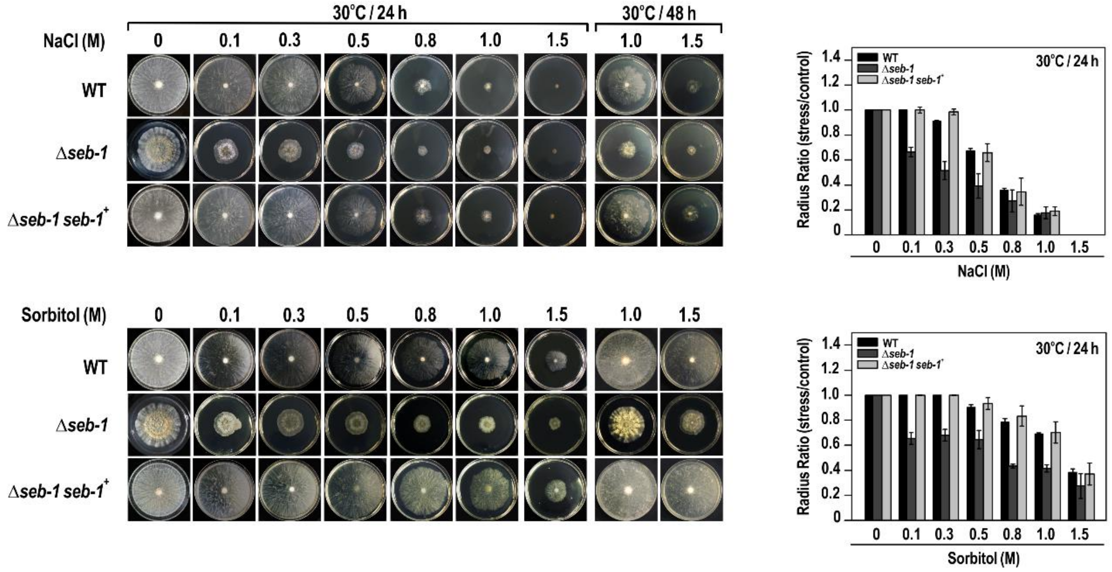
30°C / 24 h
30°C / 48 h
NaCl (M)
0
0.1
0.3
0.5
0.8
1.0
1.5
WT
Δseb-1
Δseb-1 seb-1+
Sorbitol (M)
Radius Ratio (stress/control)
Δseb-1 seb-1+

B

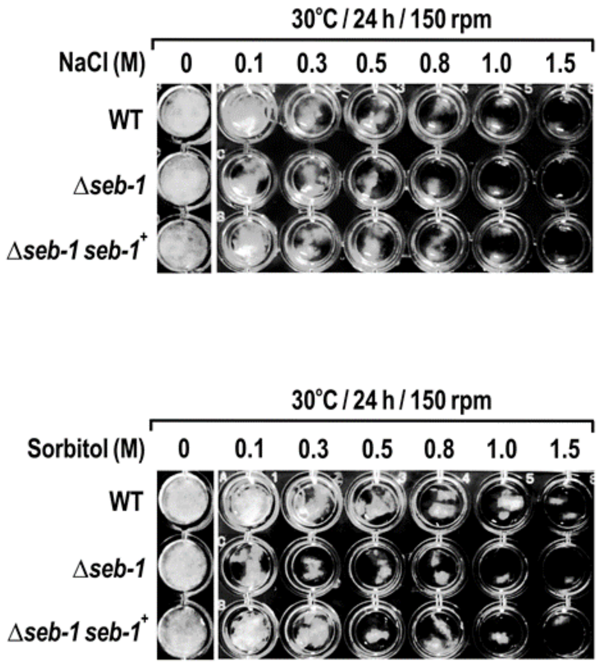
30°C / 24 h / 150 rpm
NaCl (M)
0
0.1
0.3
0.5
0.8
1.0
1.5
WT
Δseb-1
Δseb-1 seb-1+
Sorbitol (M)

Figure S3. The Δ*seb-1* strain is sensitive to high osmolarity. (A) The wild-type, Δ*seb-1*, and Δ*seb-1* complemented strains were inoculated on solid VM medium containing increased concentrations of NaCl (0.1 to 1.5 M) and sorbitol (0.1 to 1.5 M) and cultivated at 30ºC for 24 and 48 h. (B) Growth of the strains was evaluated in liquid VM medium containing varying concentrations of NaCl and sorbitol. WT: wild-type strain; Δ*seb-1*: strain mutated in the ORF NCU02671; Δ*seb-1 seb-1*$^{+}$: Δ*seb-1* complemented strain (Δ*seb-1 his-3::Pccg-1-seb1-sfgfp*).
