# Supplementary material for: The SEB-1 Transcription Factor Binds to the STRE Motif in Neurospora crassa and Regulates a Variety of Cellular Processes Including the Stress Response and Reserve Carbohydrate Metabolism
Source: G3 (Bethesda). 2016 Mar 16;6(5):1327–43. doi: 10.1534/g3.116.028506 (PMC4856084; doi:10.1534/g3.116.028506)
Supplement: Supplemental Material [file supp_g3.116.028506_TableS1.pdf]

Table S1. Oligonucleotides used in this work.

Primer	Sequence	Source***	Position#
*Pulldown assays and EMSA**			
bioSTRE1-1F	5'-/BIO/gtaatacgactcTTCA**AGGGG**GATTCA**AGGGG**GAT-3'	*pgsn*	-
bioSTRE1-1R	5'-/BIO/gtaatacgactcGTATTCA**AGGGGGA**TCCC-3'	*pgsn*	-
STRE1-2R	5'-A**T CCCC**CTTGAA**TCCCC**CTTGAAgagtcgtattac-3'	*pgsn*	-
STRE1-F	5'-GGGATC**CCCCT**TGAATAC-3'	*pgsn*	-1496 to -1513
STRE1-R	5'-GTATTCA**AGGGG**GATCCC-3'	*pgsn*	-1496 to -1513
*seb-1 cloning and Δseb-1 complementation***			
SEB1-F	5'-CAT**ATG**GAAGACATCATGGCTCAG-3'	NCU02671	+1 to +21
SEB1-R	5'-GAATTC**TCA**CTCGCCTCTCCTCCG-3'	NCU02671	+1999 to +2023
CsfGFPseb1-F	5'-CTAGTCTAGACAAAAC**ATG**GAAGACATCATGGCTC-3'	NCU02671	+1 to +19
CsfGFPseb1-F	5'-CCGCTTAAT**TCA**tcctcctcctcctcctccCTCGCCTCTCCTCCGCTTCTC-3'	NCU02671	+1999 to +2023
Real-time PCR			
qGNN-F	5'-ACAAGCACCCGAACCCAC-3'	NCU06698	+1134 to +1151
qGNN-R	5'-AAGGGTGGGCGATGCTGT-3'	NCU06698	+1234 to +1251
qGSN-F	5'-TACCAAGCATCACCACCAACCTCT-3'	NCU06687	+1541 to +1564
qGSN-R	5'-TGTCTGCGGCTCTTCTGGGTAAAT-3'	NCU06687	+1689 to +1712
qGPN-F	5'-TGCCAATATCGAAATCACCCGCGA-3'	NCU07027	+2247 to +2061
qGPN-R	5'-CTCGATGGCCTCAAACACCTTGA-3'	NCU07027	+2375 to +2398
qGDN-F	5'-GCGCGGTAATCAAGCCA-3'	NCU00743	+3779 to +3796
qGDN-R	5'-TGAATTTGCCGGCTTCGT-3'	NCU00743	+3935 to +3952
qGBN-F	5'-TCTGCGATGCCGAGTTGT-3'	NCU05429	+1487 to +1504
qGBN-R	5'-ACTCGTTGCCCTCGAAGT-3'	NCU05429	+1616 to +1633
qSEB1-F	5'-TTGCAACCGTCGTTTCCG-3'	NCU02671	+1657 to +1674
qSEB1-R	5'-AACTTCTTGCCGCACTCG-3'	NCU02671	+1741 to +1758

4173ACT-F	5'-CCATGTACCCTGGTCTCTCCGAC-3'	NCU04173	+1457 to +1479
4173ACT-R	5'-CCACCGATCCAGACGGAGTACTTG-3'	NCU04173	+1551 to +1574
ChIP-qPCR			
emsaSTRE1-F	5'-CACTGCACAGATCTGGAGGACC-3'	p*gsn*	-1590 to -1569
emsaSTRE1-R	5'-TCTTGAGACATCCATGGGCATTTCAG-3'	p*gsn*	-1433 to -1408
STRE2i-F	5'-GCTTCAGTGAGGCCCCGT-3'	p*gsn*	-350 to -333
STRE2i-R	5'-GCAGATCAGGTCGACGTAGCC-3'	p*gsn*	-205 to -185
pGPNNit-F2	5'-CTGGCTGGCTCCGTCTTA-3'	p*gpn*	-725 to -704
pGPNNit-R2	5'-GAGGTAAGTGGGGCAGTC-3'	p*gpn*	-527 to -510
GNNp-F2	5'-CGGCCGTTGTTCCATTCA-3'	p*gnn*	-954 to -937
GNNp-R3	5'-CAACCACCCGGACGAGGAC-3'	p*gnn*	-776 to -758
BRANCH-FP3	5'-GCCCCTCCATGAAGCGAAGA-3'	p*gbn*	-1215 to -1196
BRANCH-RP1	5'-TGGTTGGGCTTCTGGGCG-3'	p*gbn*	-1133 to -1116
DEBp-F2	5'-GCCTGTTTTCTGACGGGT-3'	p*gdn*	-707 to -690
DEBp-R2	5'-TTGGCTGTGATAGGACCG-3'	p*gdn*	-551 to -534
qUbi-F	5'-CGAGTCTTCGGATACGATTG-3'	NCU05995	+735 to +754
qUbi-R	5'-CCATCCTCCAACTGCTTAC-3'	NCU05995	+842 to +824

*The STRE1 motifs are represented in bold and the spacer regions in lower cases. The oligonucleotides mSTRE1-F and mSTRE1-R contain the mutated STRE1 core sequence represented in bold. BIO - biotin.

**The ORF NCU02671 ATG start codon and TCA stop codon are represented in bold, respectively, in the SEB-1-F/R and CsfGFPseb1-F/R oligonucleotides. The underlined sequence in the oligonucleotide CsfGFPseb1-F is the *N. crassa* Kozak sequence. The nucleotide sequence encoding 6 glycines is represented in lower cases.

***ORFs NCU06687 (*gsn*, glycogen synthase), NCU05429 (*gbn*, 1,4-α-glucan branching enzyme), NCU06698 (*gnn*, glycogenin), NCU07027 (*gpn*, glycogen phosphorylase), NCU00743 (*gdn*, glycogen debranching enzyme), NCU02671 (*seb-1*, SEB-1 zinc finger transcription factor), NCU05995 (*ubi*, ubiquitin). p*gnn*, p*gsn,* p*gbn,* p*gpn,* and p*gdn*-promoter of the genes *gnn, gsn, gbn, gpn,* and *gdn*, respectively.

#The DNA oligonucleotides are positioned according to the ATG start site.
